# Relaxed random walk model coupled with ecological niche modeling unravel the dispersal dynamics of a Neotropical savanna tree species in the deeper Quaternary

**DOI:** 10.3389/fpls.2015.00653

**Published:** 2015-08-25

**Authors:** Rosane G. Collevatti, Levi C. Terribile, Suelen G. Rabelo, Matheus S. Lima-Ribeiro

**Affiliations:** ^1^Laboratório de Genética & Biodiversidade, Instituto de Ciencias Biológicas, Universidade Federal de GoiásGoiânia, Brasil; ^2^Laboratório de Macroecologia, Universidade Federal de GoiásJataí, Brasil

**Keywords:** Bignoniaceae, brownian diffusion, coalescent simulation, phylogeography, Quaternary climate changes, *Tabebuia aurea*

## Abstract

Understanding the dispersal routes of Neotropical savanna tree species is an essential step to unravel the effects of past climate change on genetic patterns, species distribution and population demography. Here we reconstruct the demographic history and dispersal dynamics of the Neotropical savanna tree species *Tabebuia aurea* to understand the effects of Quaternary climate change on its current spatial patterns of genetic diversity. We sampled 285 individuals from 21 populations throughout Brazilian savannas and sequenced all individuals for three chloroplast intergenic spacers and ITS nrDNA. We analyzed data using a multi-model inference framework by coupling the relaxed random walk model (RRW), ecological niche modeling (ENM) and statistical phylogeography. The most recent common ancestor of *T. aurea* lineages dated from ~4.0 ± 2.5 Ma. *T. aurea* lineages cyclically dispersed from the West toward the Central-West Brazil, and from the Southeast toward the East and Northeast Brazil, following the paleodistribution dynamics shown by the ENMs through the last glacial cycle. A historical refugium through time may have allowed dispersal of lineages among populations of Central Brazil, overlapping with population expansion during interglacial periods and the diversification of new lineages. Range and population expansion through the Quaternary were, respectively, the most frequent prediction from ENMs and the most likely demographic scenario from coalescent simulations. Consistent phylogeographic patterns among multiple modeling inferences indicate a promising approach, allowing us to understand how cyclical climate changes through the Quaternary drove complex population dynamics and the current patterns of species distribution and genetic diversity.

## Introduction

Spatial displacements in species distributions due to Quaternary climate changes have played an important role in shaping the genetic diversity of many species across space (e.g., Taberlet et al., 1998). Postglacial range expansion, for instance, may have led to spatial genome assortment due to leading edge colonization as the species tracks suitable environments (Hewitt, 1996). The spreading from the leading edge may lead to bottlenecks of the colonizing genome, decreasing genetic diversity in some new colonizing areas. In addition, allele surfing, i.e., the spread and frequency increase of a low-frequency allele that migrates on the wave of advance of a population in expansion (Excoffier and Ray, 2008; Arenas et al., 2012), and density-dependent processes due to the fast colonization and founder events may also cause patches and sectoring in genetic diversity (see Excoffier et al., 2009; Waters et al., 2013 for reviews). Investigating these aspects of paleodistribution dynamics has been a key point to understand the effects of past colonization and to unravel the role of Quaternary climate changes in shaping the current spatial pattern of genetic diversity worldwide (e.g., Petit et al., 2002).

A recurrent approach to reconstruct past species distributions and generate independent paleoscenarios of demographical history have been coupling ecological niche modeling (ENM) with fossil data and paleoclimatic simulations in an ecological and biogeographical context (e.g., Lima et al., 2014; Metcalf et al., 2014). Subsequently, the alternative hypotheses of demographical histories are tested using coalescence analysis (e.g., Carstens and Richards, 2007; Knowles et al., 2007; Collevatti et al., 2013a). In the context of multi-model inference, ENM and coalescence modeling have provided a promising and valuable tool by considering important sources of uncertainty during historical reconstructions, such as ENM algorithms and paleoclimatic simulations uncertainties (Collevatti et al., 2012a,b), and stochastic variance in coalescent models (Kuhner, 2008). However, such an approach does not directly recover the spatio-temporal lineage dispersal process, which has a central role on the evolutionary dynamics and structure of populations. For instance, spatial inferences about dispersal events are often limited to the indirect interpretation of evolutionary histories by analyzing the predicted shifts in species' geographical range over time and the spatial locations of the sampled populations. As a consequence, the source, pathways, and routes of migration remain uncertain, and indirect inferences limit the scope of hypotheses that could be tested in phylogeography. Thus, together with ENM and coalescence modeling, we propose that a direct spatio-temporal reconstruction of lineage dispersal (Lemey et al., 2009, 2010) should integrate the context of multi-model inference to better understand the climate footprint on demographic history, shaping the spatial pattern in genetic diversity (see also Collevatti et al., 2015).

A multi-model inference approach is particularly useful to investigate the complex dynamics and current patterns of genetic diversity in tree species with a long generation time and life span. This is the case of *Tabebuia aurea* (Bignoniaceae), which is widely distributed through the Neotropics, occurring in seasonal savannas and wet-savanna grasslands (Figure S1 in Appendix S2). Locally it is distributed in well-delimited patches with high density. The species is hermaphroditic and pollinated mainly by large-size bees such as bumblebees (*Bombus* spp.), carpenter bees (*Xylocopa* spp.) and *Centris* spp., and its winged seeds are wind dispersed.

During the Last Glacial Maximum (LGM), for instance, the climate was drier in most of South America, leading to a retraction in geographical range of many arboreal savanna taxa and expansion of grasslands (Salgado-Labouriau, 1997; Behling, 2003). Because glaciations were recurrent throughout the Quaternary, the cycles of range retraction and expansion during glacial and interglacial phases may have left genetic signatures in Neotropical savannas species (e.g., Collevatti et al., 2012b). Moreover, understanding historical processes like dispersal routes in the Neotropics is often compromised because of the lack of fossil records for most species (but see an example in Lima et al., 2014 for a swamp palm species). Thus, integrating direct spatio-temporal reconstruction of lineage diffusion (Lemey et al., 2009, 2010) may give clues to the pathways of lineage dispersal across the Neotropics through the Quaternary.

In this study, we reconstructed the demographical history and dispersal dynamics of the Neotropical savanna tree species *T. aurea* through the Quaternary using a multi-model inference approach to unravel the climate change effects on its current spatial pattern of genetic diversity. Because Neotropical savanna species have been showing range retraction during the LGM followed by expansion (e.g., Collevatti et al., 2012b, 2013b), we expect that *T. aurea* had a range and demographic retraction during glacial phases, leading to high population differentiation and low genetic connectivity among populations.

## Materials and methods

### Population sampling

**Figure 1:**
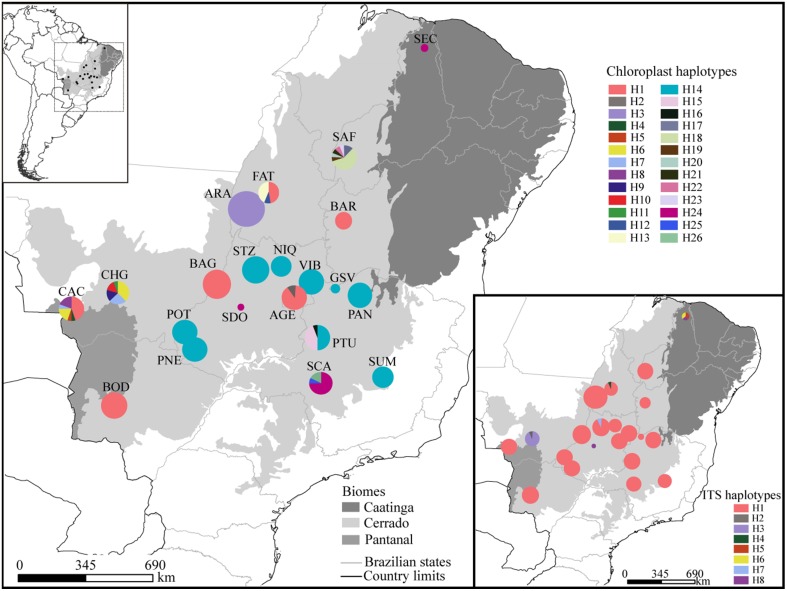
**Geographical distribution of haplotypes**. Different colors were assigned for each haplotype according to the figure legend. The circle size represents the sample size in each population and the circle sections represent the haplotype frequency in each sampled population. For details on population codes and localities see Table S1 in Appendix S1.

We sampled 21 populations (285 individuals) of *T*. *aurea* throughout the Brazilian savanna (Figure 1, see also Table S1 in Appendix S1). Distance between population pairs ranged from 69.0 to ~3800 km. We sampled only adult individuals to avoid effects of high kinship on estimation of genetic parameters. In populations with more than 16 adult individuals, we sampled and sequenced 16 individuals and in populations smaller than this we sampled all individuals (Table 1). We focused our sampling efforts on the savannas of Central-West Brazil due to the current higher abundance of *T. aurea* and our focus on savanna biogeography. In addition, although occurrence map (see Figure S1 in Appendix S2) show records of *T. aurea* in the Northeastern Brazil (Caatinga biome), populations in these places are barely found (we could found only one or two individuals in most places we visited, such as population SEC). The Northeast Brazil is dominated by a xeromorphic steppe vegetation with seasonally dry forest and some island of savanna where *T. aurea* may occur but the high level of anthropogenic disturbance had removed most natural vegetation. We also sampled and sequenced individuals of *Tabebuia impetiginosa, Tabebuia chrysotrica*, and *Cybistax antisyphillitica* to include as outgroups in coalescence analyses (Table S1).

**Table 1:** **Genetic diversity and demographic parameters for 21 populations of ***Tabebuia aurea*** in Brazil for combined cpDNA data and for ITS nrDNA**.

	**cpDNA**				**ITS**			**Combined cpDNA and ITS nrDNA**					
**Pop**	***N***	***k***	***h***	**π (*SD*)**	***k***	***h***	**π (*SD*)**	**θ**	**θ–95% interval**	***N_e_***	***N_*e*_*–95% interval**	***g***	***g*–95% interval**
AGE	16	2	0.441	0.0127 (0.0067)	1	0.000	0.0000	0.00117	0.00031–0.00218	1629.7	425.9–3020.8	114.78	−491.64 to 898.71
ARA	14	1	0.000	0.0000	1	0.000	0.0000	0.00019	0.00003–0.00038	276.2	37.2–520.3	259.48	−442.66 to 976.39
BAG	18	1	0.000	0.0000	1	0.000	0.0000	0.00012	0.00001–0.00059	165.7	14.7–819.7	398.88	−356.14 to 990.39
BAR	12	1	0.000	0.0000	1	0.000	0.0000	0.00006	0.00001–0.00014	82.8	14.7–187.7	205.66	−429.11 to 996.42
BOD	17	1	0.000	0.0000	1	0.000	0.0000	0.00005	0.00001–0.00011	68.5	14.5–155.7	288.64	−435.94 to 990.31
CAC	16	6	0.542	0.0842 (0.0428)	1	0.000	0.0000	0.00263	0.00058–0.00606	3646.5	802.1–8419.0	142.59	40.54 to 847.62
CHG	15	5	0.617	0.0334 (0.0172)	3	0.514	0.0011 (0.0001)	0.00409	0.00087–0.00735	5683.2	1209.5–10202.8	118.99	98.60 to 426.76
FAT	22	3	0.834	0.0099 (0.0053)	3	0.256	0.0319 (0.0016)	0.00277	0.00146–0.00514	3845.3	2028.2–7143.2	30.04	9.86 to 237.16
GSV	8	1	0.000	0.0000	1	0.000	0.0000	0.00062	0.00009–0.00166	865.5	135.9–2309.7	309.98	−479.87 to 895.55
NIQ	14	1	0.000	0.0000	1	0.000	0.0000	0.00006	0.00001–0.00027	76.8	14.9–367.9	116.62	−496.46 to 936.36
PAN	16	1	0.000	0.0000	1	0.000	0.0000	0.00011	0.00001–0.00028	148.9	14.8–394.2	197.63	−487.72 to 902.18
PNE	16	1	0.000	0.0000	1	0.000	0.0000	0.00027	0.00004–0.00059	378.3	48.9–820.2	−328.35	−498.69 to −57.59
POT	16	1	0.000	0.0000	1	0.000	0.0000	0.00004	0.00001–0.00009	60.4	14.0–129.9	264.05	−468.77 to 936.29
PTU	16	3	0.792	0.0274 (0.0141)	1	0.000	0.0000	0.00230	0.00107–0.00488	3199.9	1482.5–6782.5	90.38	3.53 to 204.53
SAF	16	7	0.771	0.0137 (0.0072)	1	0.000	0.0000	0.01660	0.00811–0.02999	23106.9	11268.5–41533.3	168.56	36.96 to 280.83
SCA	15	3	0.628	0.0148 (0.0077)	1	0.000	0.0000	0.00472	0.00205–0.00882	6561.8	2850.7–12254.4	338.21	9.09 to 752.49
SDO	03	1	0.000	0.0000	1	0.000	0.0000	–	–	–	–	–	–
SEC	04	1	0.000	0.0000	3	0.500	0.0019 (0.0003)	–	–	–	–	–	–
SUM	14	1	0.000	0.0000	1	0.000	0.0000	0.00149	0.00068–0.00234	2070.7	947.8–3245.8	−30.15	−98.82 to 41.06
STZ	17	1	0.000	0.0000	2	0.471	0.0009 (0.0001)	0.00024	0.00004–0.00054	334.9	53.3–753.6	96.21	−498.38 to 920.54
VIB	16	1	0.000	0.0000	2	0.125	0.0001 (0.0000)	0.00024	0.00005–0.00054	333.5	70.6–747.7	−269.59	−419.58 to −98.29
Mean	–	2.1	0.220	0.0093 (0.0197)	1.4	0.089	0.0006 (0.0025)			2537.4			
SD	–	1.9	0.329	–	0.4	0.158	–			5103.8			
Overall	285	26	0.933	0.0466 (0.0223)	8	0.267	0.0186 (0.0096)	0.02940	0.02430–0.03510	40788.9	33813.9–48715.3	70.49	7.22 to 822.84

*N, sample size; k, number of haplotypes; h, haplotype diversity; π, nucleotide diversity; SD, standard deviation; θ, coalescent parameter; N_e_, effective population size; g, exponential growth parameter (all values are not significantly different from zero based on the credibility interval around the estimates); 95% IC is the credibility interval at 95%*.

### Genetic data

To generate genetic data, we sequenced three intergenic spacers of chloroplast DNA (cpDNA): *psbA-trnH, trnC*-*ycf6*, and *trnS*-*trnG* (Shaw et al., 2005) and the region ITS1 + 5.8S + ITS2 (ITS hereafter) from nuclear ribosomal DNA (nrDNA) (Desfeux and Lejeune, 1996). PCR conditions, amplifications and sequencing followed Collevatti et al. (2012a).

Consensus sequences were obtained using the software SeqScape v2.6 (Applied Biosystems, CA) and aligned with the software ClustalΩ (Sievers et al., 2011). Polymorphisms at mononucleotide microsatellites were excluded due to ambiguous alignment and to higher mutation rates. Long indels (usually with more than 5 bp) were coded as one evolutionary event (one character).

### Genetic diversity and structure of populations

To investigate genetic diversity and structure of populations, chloroplast and nuclear ITS were analyzed separately. Nucleotide (π) and haplotype (*h*) diversity were estimated for each population and overall populations following Nei (1987) using the software ArlequinVer 3.11 (Excoffier et al., 2005). To understand the phylogenetic relationships among haplotypes, intraspecific phylogenies for chloroplast and ITS data were inferred using median-joining network analysis implemented in the software Network 4.6.1.0 (Forster et al., 2004). To test the hypothesis of population differentiation, we performed an analysis of molecular variance (AMOVA, Excoffier et al., 1992) and estimated *F*_*ST*_ and tested whether genetic differentiation across *T. aurea* populations (linearized *F*_*ST*_) is correlated with geographical distance (logarithm) among populations using the Mantel test. Analyses were performed using the software ArlequinVer 3.11 and statistical significance was tested by a non-parametric permutation test (10,000 permutations).

### Phylogeographic reconstruction

#### Population demography

All coalescent analyses were performed with chloroplast and nrDNA ITS partitions concatenated, but giving separate priors. No evidence of heterozygous individuals was found when ITS sequences were analyzed using SeqScape v2.6 (Applied Biosystems. CA). Thus, recombination was neglected in all coalescent analyses. To set the priors, evolutionary model selection for both chloroplast and ITS regions was performed using Akaike Information Criterion implemented in the software jModelTest2 (Darriba et al., 2012). For chloroplast regions, the model HKY+G was selected, with gamma shape equal to 1.87. For ITS, the evolutionary model JC was selected.

To study the dynamics in effective population size and genetic connectivity among populations, we estimated the mutation or coalescent parameter theta (θ = 2 μN_*e*_, for haploid genome, θ = 4 μN_*e*_, for diploid genome), the exponential growth rate (*g*, where θ_*t*_ = θ_*now*_ exp(−*gtμ*) and *t* is time in mutational unit), and the immigration rate among all population pairs (*M* = 2 N_*e*_m/θ, for haploid genome, *M* = 4 N_*e*_m/θ, for diploid genome). Estimations were based on Bayesian model using the Markov Chain Monte Carlo (MCMC) approach implemented in Lamarc 2.1.9 software (Kuhner, 2006). Because of the high number of populations, to estimate growth rate, we constrained migration (maintained migration constant), and migration was estimated in independent runs. Each analysis was run with 20 initial chains of 4000 steps and three final chains of 50,000 steps. The chains were sampled every 100 steps. We used the default settings for the initial estimate of theta. The program was run four times for each parameter to certify convergence among runs and validate the analyses using Tracer v1.6, and combined results were then generated. Results were considered when ESS ≥ 200 (effective sample size) and when marginal posterior probability densities were unimodal and converged among runs. Because of the low sample size, demographic parameters were not estimated for populations SDO and SEC. Effective population size was obtained from θ (Kingman, 1982) using a generation time of 12 years (based on flowering time on permanent plots; RG Collevatti, unpublished data).

Then, we performed a Coalescent Extended Bayesian Skyline Plot (EBSP) analysis (Heled and Drummond, 2008) implemented in BEAST 1.8.0 (Drummond and Rambaut, 2007) which calculates the effective population size (*N*_*e*_) through time to better understand changes in population size, combining data from different partitions. We used the substitution models reported above and the relaxed molecular clock model (uncorrelated lognormal) for both chloroplast and ITS. Mutation rates for both chloroplast and ITS regions were the same used for a taxonomic related species *Tabebuia impetiginosa* (Collevatti et al., 2012a). Four independent analyses were run for 30 million generations. Convergence and stationarity were checked, and the independent runs were combined using the software Tracer v1.6. Results were considered when ESS ≥ 200. We also inspected lineage (haplotype) diversification using Lineage Through Time (LTT) analysis to provide clues of major divergence timing. We performed the Fu's *FS* tests of neutrality implemented in Arlequin 3.11. Negative values for *FS* indicate an excess of rare alleles or new mutations in the genealogy resulting from either population expansion or selective sweeps.

#### Lineages dispersal

To reconstruct the spatio-temporal history of lineage dispersal, we used the relaxed random walk model (RRW, Lemey et al., 2009, 2010) implemented in the software BEAST that analyses molecular sequence evolution, demographic model, and lineage dispersal in space and time simultaneously. RRW infers continuous phylogeographical diffusion while simultaneously reconstructing the evolutionary history in time (Lemey et al., 2010). More specifically, the approach identifies the phylogeographical diffusion processes by stochastically selecting a diffusion rate scalar on each branch of the rooted phylogeny from an underlying discretized rate distribution while running a MCMC. Because this framework is based on stochastic models, it naturally accesses the uncertainties along the ancestral state reconstructions and the underlying phylogeographical process (Lemey et al., 2009, 2010).

To build RRW, we used both sequence partitions with unlinked priors but sharing the same location rate matrix (Lemey et al., 2009, 2010), including the rate for Bayesian Stochastic Search Variable Selection procedure (BSSVS), which considers a limited number of rates (at least k–1) to explain the phylogenetic diffusion process. The sampling locality was added as discrete character states (*k* = 21 localities). For the diffusion process, we used the Symmetric Substitution Model that uses a standard continuous-time Markov chain (CTMC) in which the transition rates between locations are reversible. Priors for sequence evolution were the same as described above. For tree priors, we used the Coalescent GMRF Bayesian Skyride model (Minin et al., 2008) and for location state rate the prior CTMC Rate Reference (Ferreira and Suchard, 2008). Four runs were performed with 30 million generations, and stability was analyzed using Tracer 1.6, and results were considered when ESS ≥ 200. The annotate tree (maximum clade credibility tree) was generated with 10% of burnin. The spatio-temporal reconstruction was performed using SPREAD 1.0.6 (Bielejec et al., 2011). We also analyzed the well-supported transition rates using the Bayes factors (BF) test implemented in SPREAD. Transitions rates between localities were considered only for BF > 8.0.

#### Coalescent tree and time to most recent common ancestor

We obtained a coalescent tree based on Bayesian coalescent analysis implemented in the software BEAST 1.8.0 to estimate time to most recent common ancestor (TMRCA). For this analysis, we included the outgroup sequences from *T. impetiginosa, T. chrysotrica*, and *C. antisyphillitica*. We used the same priors of EBSP, except that for tree prior we used Coalescent Expansion Growth based on the results of EBSP and ENM (see Results below). MCMC conditions and number of runs also remained unchanged. The independent runs were analyzed using Tracer 1.6 and results were considered when ESS ≥ 200. We also ran an empty alignment (sampling only from priors) to verify the sensitivity of results to the given priors. The analysis showed that our data is informative because posterior values (e.g., posterior probability) were different from those obtained from empty alignment.

### Ecological niche models

#### Occurrence records and environmental layers

We obtained 237 occurrence records of *T. aurea* across Neotropics from the online databases GBIF (Global Biodiversity Information Facility http://www.gbif.org/) and Species Link (http://splink.cria.org.br/), which were mapped in a grid of cells of 0.5° × 0.5° (longitude × latitude), corresponding to 55 km at the equator, encompassing the entire Neotropics (Table S2 in Appendix S1, Figure S1 in Appendix S2).

The environmental layers were represented by five bioclimatic variables (annual mean temperature, mean diurnal range, isothermality—mean diurnal range/temperature annual range, precipitation of wettest month, and precipitation of driest month), selected by factorial analysis with Varimax rotation from 19 bioclimatic variables obtained in the EcoClimate database (www.ecoclimate.org), along with subsoil pH (30–100 cm; from Harmonized World Soil Database—version 1.1, FAO/IIASA/ISRIC/ISS-CAS/JR, 2009). We assume subsoil pH to be constant between LGM and pre-industrial, which was used in ENMs as a “constraint variable” to better model the environmental preferences of *T. aurea*. The bioclimatic variables were obtained for pre-industrial (representing current climate conditions), mid-Holocene (6 ka) and Last Glacial Maximum (LGM; 21 ka) from four coupled Atmosphere-Ocean General Circulation Models (AOGCM): CCSM4, CNRM-CM5, MIROC-ESM, and MRI-CGCM3 (Table S3).

#### Palaeodistribution modeling

Estimates of the current and past potential distribution of *T. aurea* were obtained using 13 algorithms by modeling its ecological niche (Table S4). When needed, we randomly selected pseudo-absences across the Neotropical grid cells keeping prevalence equal to 0.5 (see Stokland et al., 2011). The ENMs were then built on current climatic scenario and projected onto the climatic conditions during both the mid-Holocene (6 ka) and LGM (21 ka). All ENM procedures were run in the integrated computational platform BIOENSEMBLES, which provides predictions based on the ensemble approach (see Araújo and New, 2006; Diniz-Filho et al., 2009).

For each algorithm and AOGCM, models were built 50 times. After eliminating the models with poor performance (True Skill Statistics—TSS - < 0.5, Allouche et al., 2006), we computed the consensus maps using thresholds established by the ROC curve and the frequency of occurrence was used here as a measure of suitability for *T. aurea* across the Neotropical grid cells.

Next, we applied a hierarchical ANOVA using the predicted suitability from all models (13 ENMs × 4 AOGCMs × 3 Times) as a response variable to disentangle the effects of climate change on species distribution through the time from predictive uncertainties in the potential distribution due to modeling components (i.e., ENMs, AOGCMs). For this, the ENM and AOGCM components were nested into the time component, but crossed by a two-way factorial design within each time period (see Terribile et al., 2012 for details about hierarchical design). Because we do not have repetitions, the residual term from ANOVA represents the interaction between AOGCM and ENM (AOGCM × ENM; Sokal and Rohlf, 1995). Finally, the full ensemble was obtained for each time period using the predictive performances (TSS) to compute a weighted mean of suitabilities (Table S5), from which historical refugium were mapped (i.e., all grid cells with suitability values ≥0.5 during the three time periods). The threshold of 0.5 corresponds to the 10th percentile of the distribution of suitability values related to the species occurrence records, i.e., we excluded the lowest 10% suitability values from the species occurrence records. We performed sensitivity analyses using different thresholds, 0.4, 0.6, and 0.7, corresponding to the 5th, 12th, and 17th percentiles, respectively, and the results were virtually the same as using threshold 0.5. The result differed only for a threshold >0.7, resulting in a small refugium in central-west Brazil.

#### Setting demographic scenarios

The ENMs resulted in 52 predictive maps (13 ENMs × 4 AOGCMs) for each time period, which were used to support alternative demographic scenarios. By keeping constant the ENM and AOGCM across time periods, we computed the range shift across the last glacial cycle (i.e., the difference in predicted range size between current and LGM—21 ka), and classified the 52 predictive maps according to three general demographic scenarios: (i) “Range Stability,” no difference in range size through time; (ii) “Range Expansion,” range size was lower at LGM than in present-day; (iii) “Range Retraction,” range size was larger at LGM than in present-day. Map classification for coalescent simulation was performed only for the time slice from LGM to present-day due to the TMRCA for the analyzed genome regions (~4.4 Ma; see results below).

Along with the three hypotheses supported by ENMs (see above), a fourth biogeographic hypothesis of “Multiple Refugia” was derived from paleoclimatic (e.g., Ab'sáber, 2000) and paleovegetational reconstructions (e.g., Salgado-Labouriau, 1997).

### Demographic history simulation

The demographic scenarios were simulated based on coalescent analysis (Kingman, 1982) implemented in the software ByeSSC (Excoffier et al., 2000), following the framework described in Collevatti et al. (2012a, 2013b). For model calibration, we used the demographic parameters estimated with Lamarc software and the same priors for sequence evolution used in Bayesian analyses (see above). The number of generations until LGM (21 ka) was calculated using the generation time of *T. aurea* (12 years, RG Collevatti, unpublished data).

**Figure 2:**
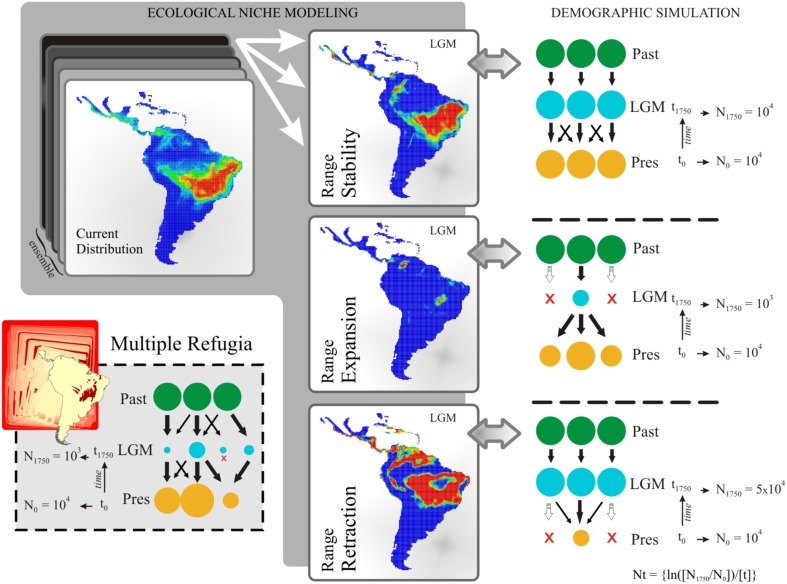
**Schematic representation of the demographic history scenarios simulated for ***Tabebuia aurea*** and their paleodistribution representations**. Circles represent hypothetical demes and indicate population stability or shrinkage through the time. LGM, last glacial maximum; Pres, present-day; N0 and N1750, effective population size at time t0 (present-day) and time t1750 (1750 generations ago, matching LGM), respectively; Nt, logarithm function for effective population size variation in coalescent simulation. The migration rate was 0.01/generation.

Population dynamics were simulated backwards, with 21 demes, from t0 (present) to t1750 generations ago (at 21 ka), with sizes Nt = {ln(N1/N0)/t}. At t0, all demes had the same population size N0 and reached N1750 according to our theoretical expectation for each demographic scenario (see Figure 2 for details). Given the high variation in *T. aurea* effective population sizes (see Table 1), we performed simulations with different initial deme sizes, N0 = 100, N0 = 1000, and N0 = 10,000 for all scenarios. Because coalescent simulations run backward through time, negative growth implies population expansion from the past, and a positive growth a population smaller now than in the past. The demographic scenario predicted by “Range Expansion” was then simulated by applying an exponentially negative population growth from present to 21 ka, reaching N1750 = 1000, if N0 = 10,000, or N1750 = 100, if N0 = 1000, and N1750 = 10, if N0 = 100. In opposition, the “Range Retraction” scenario was simulated applying exponentially positive population growth during the same period, attaining N1750 = 50,000, if N0 = 10,000, or N1750 = 5000, if N0 = 1000 and N1750 = 500, if N0 = 100. Migration was simulated considering all current deme descendants from lineages originally in deme 1 at t generations ago, meaning that while the coalescent tree builds back through time, there is a 0.01/generation chance that each lineage in deme x will migrate to deme 1. We also simulated different values of migration rate. Values < 0.01 were not sufficient to show any demographic variation at the time scale we are working and values >0.1 retrieved equal likelihoods for all models. For the “Range Retraction” hypothesis, we considered that each lineage in deme x will migrate to deme 1 and then shrink until extinction. For the “Multiple Refugia” scenario we considered a finite island model; i.e., all current populations are descendant from lineages originally in the demes at t1750 generations ago, meaning that while the tree builds back through time, there is a 0.01/generation chance that each lineage will migrate to deme x.

The simulated haplotype and nucleotide diversities for the four alternative demographic scenarios, across 2000 simulations, were compared with the empirical haplotype and nucleotide diversity (mean for the 21 populations). One-tailed probability (*P*) and Akaike Information Criterion (*AIC*) were estimated for each comparison. The log-likelihood was estimated as the product of the height of the empirical frequency distribution at the observed value of diversity by the maximum height of the distribution (see BayeSSC website www.stanford.edu/group/hadlylab/ssc/index.html). *AIC* was transformed into *AIC* weight of evidence (*AICw*), given by exp[−0.5(*AIC* − *AIC*min)] (see Burnham and Anderson, 2002), from which we obtained Δ*AIC*; i.e., the difference of AICw between each model and the best model. Models with ΔAIC < 2 were considered as equally plausible to explain the observed pattern (Zuur et al., 2009).

## Results

### Genetic diversity and structure of populations

**Figure 3:**
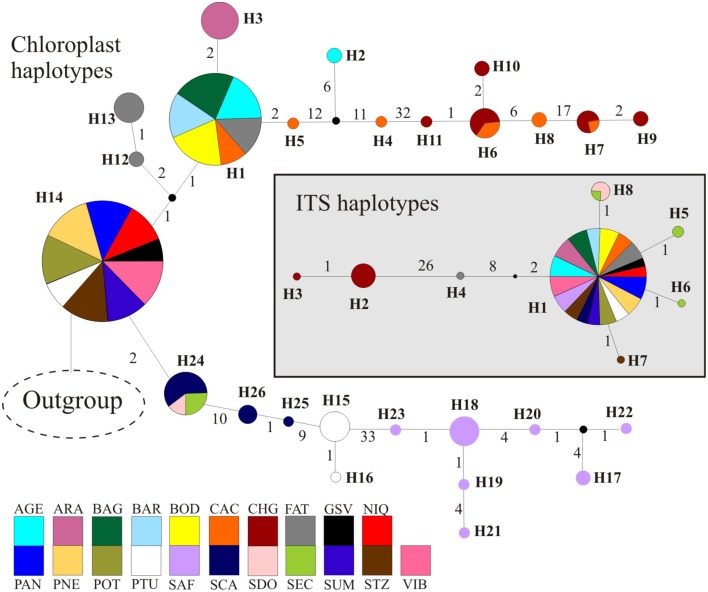
**Phylogenetic relationships among haplotypes using median-joining network**. Circumference size is proportional to the haplotype frequency. Number of mutations is shown along lines in the network; small black circles are the median vectors. Different colors were assigned for each population according to the figure legend.

The combined data of chloroplast intergenic spacers generated 2330 bp and the nrDNA ITS, 567 bp (see Appendix S3 for GenBank accession numbers), resulting in 2627 bp when microsatellites and ambiguous alignment were eliminated (2100 bp for chloroplast and 527 for ITS). Although 26 different chloroplast and 8 ITS haplotypes were found for the 285 individuals of *T. aurea*, most populations presented low haplotype and nucleotide diversities (Table 1). Two chloroplast haplotypes were very widespread (Figures 1, 3), H1 and H14, and one ITS haplotype (H1) was found in all populations, but CHG and SDO (Figures 1, 3). The analysis of molecular variance showed significant genetic differentiation among populations for both cpDNA (*F*_*ST*_ = 0.773; *p* < 0.001) and nuclear ITS (*F*_*ST*_ = 0.966; *p* < 0.001). Although pairwise genetic differentiation was significant among all pairs of populations for cpDNA, differentiation for nuclear ITS was mainly due to populations CHG, SDO, and SEC (Table S6). The Mantel test showed weak (chloroplast, *r*^2^ = 0.069, *p* = 0.026) or no association between the geographical distance and the genetic differentiation among pairs of populations (ITS, *r*^2^ = 0.032, *p* = 0.159).

### Phylogeographic reconstruction

#### Population demography and time to most recent common ancestor

Coalescent analyses performed with Lamarc software showed low values of mutation parameter θ for each and overall population (θ = 0.029, Table 1) and negligible gene flow among all population pairs (less than 1.0 migrant per generation, Tables S7, S8). Our results showed population growth through time, with positive values of *g* for some populations and overall populations (overall *g* = 70.49; Table 1).

**Figure 4:**
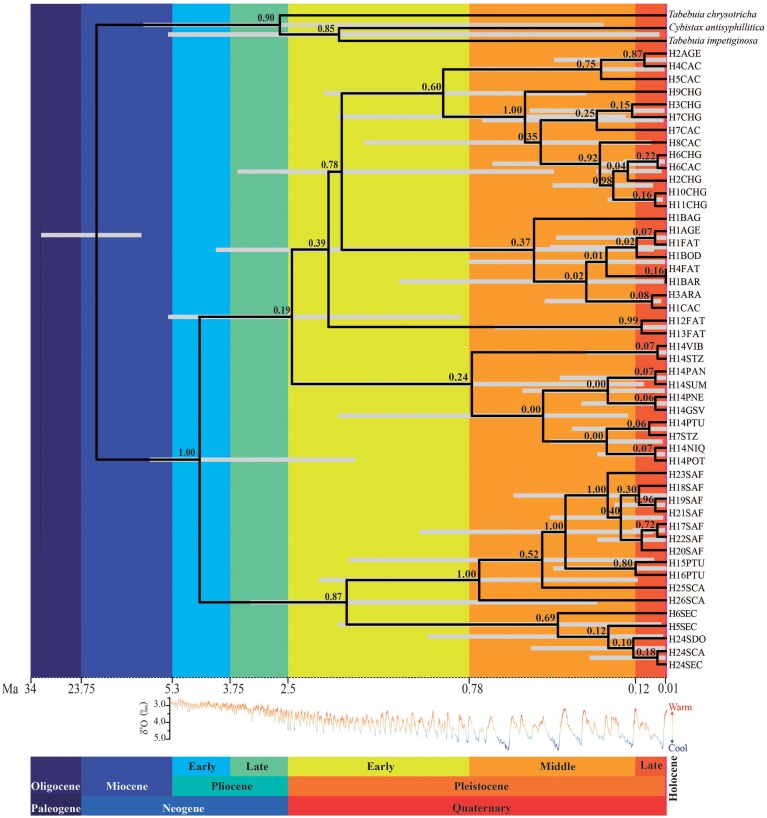
**Relationships and TMRCA of ***Tabebuia aurea*** lineages**. The light gray bar corresponds to 95% highest posterior probability of the median time to the common ancestor; the numbers above the branches are the supports to the nodes (posterior probability). The time scale is in millions of years ago (Ma). The δ^18^O curve corresponds to the composite benthic stable oxygen isotope ratios (Lisiecki and Raymo, 2005).

The number of lineages increased almost linearly with a higher rate in the last ~1.0 Ma (Figure S2). Lineages of *T. aurea* started to diverge at ~4.4 ± 2.6 Ma (Figure 4), although major divergence events dated from the Pliocene/Pleistocene transition (~2.5 ± 2.3 Ma) with increasing divergence rates after ~1.2 Ma, coinciding with the increasing in lineage diversification (Figure S2).

**Figure 5:**
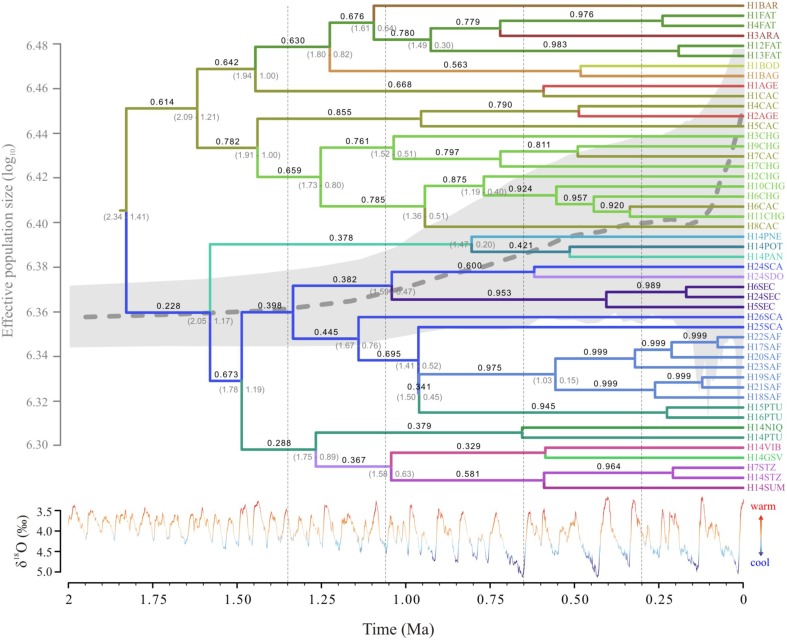
**Temporal dynamics of lineage diffusion showing the most probable locations for ***Tabebuia aurea*** lineages; branch color corresponds to the locality shown in the tip names; numbers above the branches are the location state posterior probability and below the branches are time 95% credibility intervals; the Extended Bayesian Skyline Plot is shown with the tree**. The δ^18^O curve corresponds to the composite benthic stable oxygen isotope ratios (Lisiecki and Raymo, 2005). For details on population codes and localities see Table S1 in Appendix S1 and Figure 1.

The Extended Bayesian Skyline Plot also showed increasing population growth through the last 1.2 Ma, especially in the last 100 ka (Figure 5). Fu' test was significant (*FS* = −5.978, *p* = 0.007) and potentially indicates a demographic expansion or departure from neutrality.

#### Lineages dispersal

**Figure 6:**
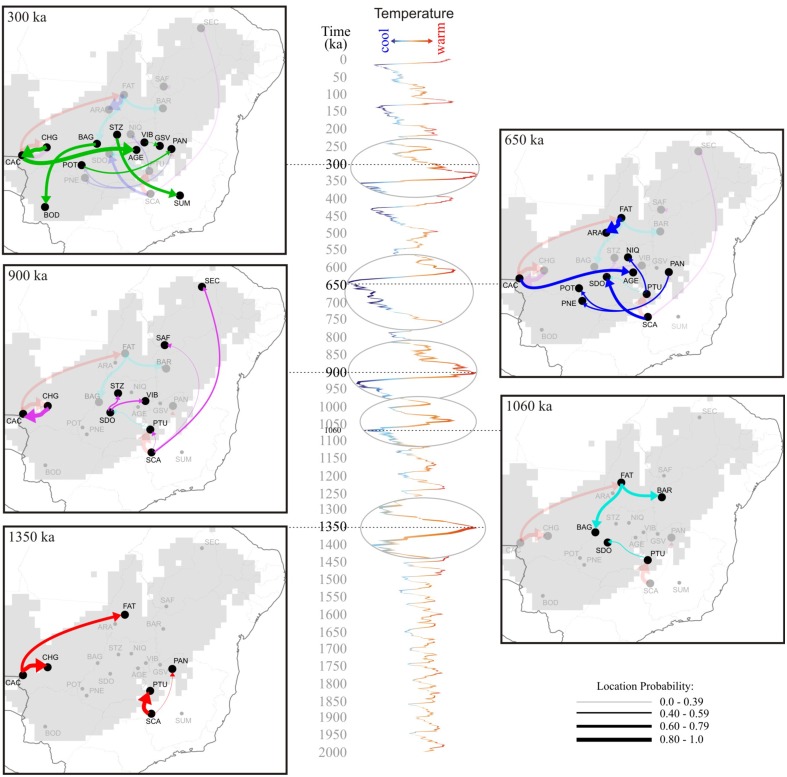
**Spatio-temporal dynamics of ***Tabebuia aurea*** lineage diffusion among the 21 populations sampled in Brazil, for 1.350 Ma, 900 ka, 650 ka, and 300 ka until present day**. Arrows between locations represent branches in the tree along which the relevant location transition occurs. The map was adapted from the .kml file provided by SPREAD software generated using Google Earth (http://earth.google.com). The δ^18^O curve corresponds to the composite benthic stable oxygen isotope ratios (Lisiecki and Raymo, 2005). For details on population codes and localities see Table S1 in Appendix S1 and Figure 1.

The phylogeographic reconstruction of lineage dispersal showed the two most probable ancestral locations for *T. aurea* lineages, one in the west (CAC) and other in the southeast Brazil (SCA) (Figure 5). These lineages started to spread at ~1.55–1.25 Ma in two main directions: from CAC, mainly to the central-west Brazil, and from SCA toward the east and northeast (Figure 6). However, most dispersal events occurred after 900 ka overlapping the temporal increase in population expansion, as shown by EBSP and the diversification timing of new lineages. From 25 dispersal events, 18 occurred during interglacial or warming periods and just 7 in glacial or cooling periods (see Figure S4). Most dispersal events during interglacial and warming periods (*n* = 14) occurred toward populations in northern Brazil, corresponding to the spatial displacement in suitable climatic conditions as showed by ENMs (see below). The mean rate of dispersal was 1.17 km/yr (*SD* = 0.978, 95% HPD interval 0.0005–3.517 km/yr), and the Bayes factor showed that most links among localities are well-supported (BF > 8.0).

### Ecological niche models

**Figure 7:**
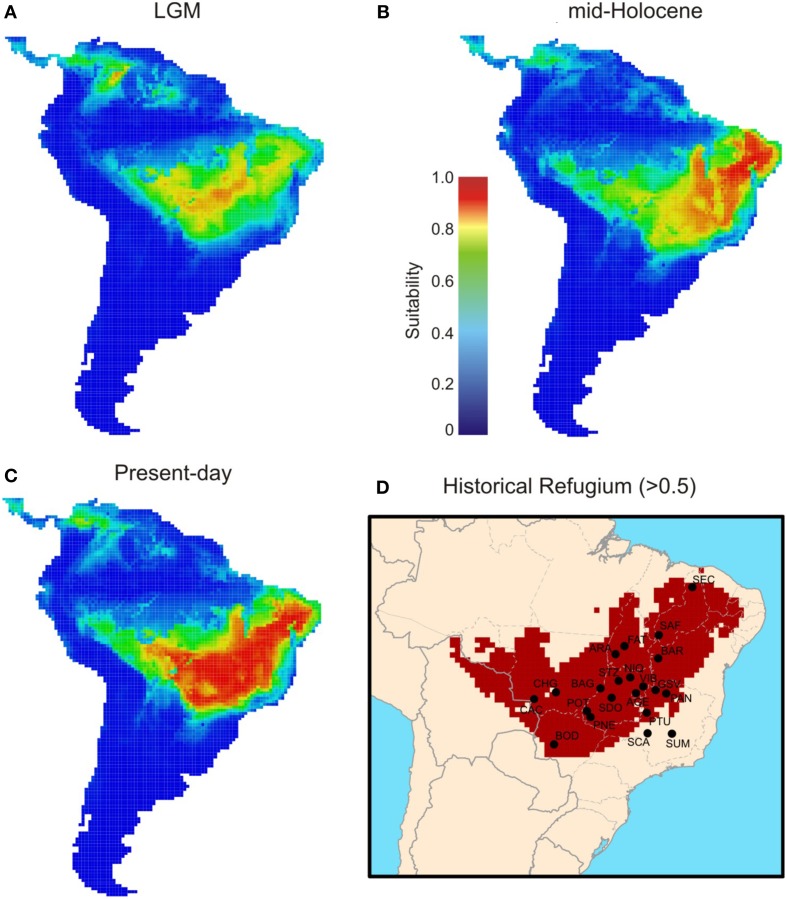
**Maps of consensus expressing the ensemble climatic suitability for ***Tabebuia aurea***, hence its predicted potential distribution across the Neotropics during the (A) LGM (21 ka), (B) mid-Holocene (6 ka), and (C) present-day**. Historical refugium **(D)** shows areas climatically suitable throughout the time.

ENMs predicted that *T. aurea* was potentially distributed across central and northeast Brazil through the last glaciation (Figure 7A). The highest levels of suitability were restricted to central Brazil, followed by a spatial displacement toward northeast Brazil during the mid-Holocene (6 ka, Figure 7B), and also expanding more recently toward the west (present-day, Figure 7C). Besides the spatial displacements, range size increased through time (see Figure S3). In addition, a wide region across central Brazil probably acted as historical refugium maintaining populations of *T. aurea* during the climate changes throughout the last glaciation (Figure 7D).

The models were well evaluated from TSS (Table S5). The analysis of uncertainty using hierarchical ANOVA showed lower proportional variance from modeling method (ENMs) than time component (Table S9, Figure S5) indicating that the ENMs were able to detect the effects of climate changes on the distribution dynamics of *T. aurea* through the last glaciation, despite the AOGCM variation.

The scenario of “Range Expansion” was the most frequent hypothesis from ENM predictions (65.3% of the 52 maps, Table 2, see also Table S10), followed by “Range Stability” (30.7%) and “Range Retraction” (4%). Moreover, the average range shift predicted by ENMs systematically follows what is expected by general demographic scenarios (Figure S6).

**Table 2:** **Comparison of the four demographic scenario models in retrieving the haplotype (h) and nucleotide (π) diversity observed for ***Tabebuia aurea***, obtained from 2000 simulations using the software BayeSSC**.

**Models**	**Chloroplast**						**ITS**						***ENM* %**
	***ΔAIC***		***AICw***		***P***		***ΔAIC***		***AICw***		***P***		
	***h***	**π**	***h***	**π**	***h***	**π**	***h***	**π**	***h***	**π**	***h***	**π**	
Stability	11.9	9.2	0.002	0.005	0.005	0.001	7.3	4.2	0.022	0.083	0.001	0.002	30.7
Retraction	–	–	–	–	–	–	–	–	–	–	–	–	4.0
Expansion	0.0	0.0	0.789	0.571	0.991	0.959	0.0	0.0	0.865	0.671	0.971	0.876	65.3
Multiple Refugia	2.7	0.5	0.209	0.424	0.435	0.354	4.1	2.3	0.113	0.243	0.743	0.623	–

*ΔAIC, the difference of AICw between each model and the best model; AICw, Akaike Information Criterion (AIC) weights; P, one-tailed probability of not rejecting the model. ENM %, the percentage of the 52 paleodistribution maps that represent each demographic scenario. See Figure 3 for details about the demographic scenarios. h, haplotype diversity; π, nucleotide diversity*.

### Demographic history simulations

Simulations using N0 = 100 presented all values of haplotype and genetic diversity lower than those observed for *T. aurea* for all demographical scenarios. Simulations using N0 = 1000 and N0 = 10,000 retrieved the same final results; i.e., the same most likely scenario, thus we show the results only for N0 = 10,000. The scenario of “Range Expansion” was the most likely hypothesis to predict the observed genetic parameters of *T. aurea*, using either one-tailed probability or *AICw* criteria (Table 2). The simulations following the “Range Retraction” scenario retrieved all values lower (see Table S11 for details) than the observed mean haplotype and nucleotide diversities for the 21 populations of *T. aurea* (Table 1), meaning that *AIC* and *P* could not be estimated. In contrast, the scenarios of “Range Stability” and “Multiple Refugia” retrieved higher values of genetic diversity than those observed for most populations (Table S11).

## Discussion

Our integrative approach shows clear convergence among phylogeographic inferences from multiple models. The reconstruction of lineages dispersal inferred using the RRW, the distribution dynamics retrieved by ENMs and the coalescent simulation predicted similar demographic and dispersal dynamics for *T. aurea* through the Quaternary.

Our results show that *T. aurea* lineages cyclically dispersed from the West toward Central-West Brazil, and from the Southeast toward the East and Northeast Brazil, following the paleodistribution dynamics showed by ENMs through the last glacial cycle. The dispersal results indicated that populations currently occurring in northern and northeastern Brazil received migrants mainly from the southwest and southeast, respectively (e.g., see the arrow direction at 1350 and 900 ka in Figure 6). Such dispersal routes have most likely occurred because suitable climatic conditions were available across a wider region during warming phases, allowing spatial displacements of the species' geographical distribution toward northeastern Brazil. Diffusion models suggested that an inverse dynamic occurred during cooling phases (e.g., 1060 and 650 ka, Figure 6), when lineages dispersed in the opposite direction, from north-northeast toward central hot and drier climate in the central-west Brazil, thus retaining more suitable niches for *T. aurea* during the cooler phases (see Collevatti et al., 2014). Yet, in intermediate warmer phases, the findings showed that lineages dispersed in both directions, mainly across central Brazil (300 ka), in response to slight changes in climatic suitability, like ENM predictions between mid-Holocene and present-day. A potential continuous historical refugium across the Neotropical savannas may be the key factor allowing uninterrupted lineage dispersal among populations of central Brazil, as predicted by the RRW (at least since the middle Pleistocene; last 900 ka). It is important to note that the dispersal of lineages already present in a population would not be detected as a new gene flow event. Thus, population connectivity could be maintained after 300 ka but our results show no dispersal of new lineages.

Concurrently, coalescent simulations showed population expansion through time (i.e., from LGM to present-day) as the most likely general scenario among alternative demographic hypotheses, matching our theoretical prediction, ENMs and extended Bayesian Skyline Plot results. Because favorable climatic conditions for *T. aurea* (i.e., hot and drier climates, see Collevatti et al., 2014) were spatially more restricted during glacial than interglacial periods in Neotropics, smaller effective population sizes during the cold phases of the last glacial cycle is a consequence of the retraction in geographical range when species spatially tracked suitable habitats. Besides, the increasing number of lineages, especially in the last ~1.25 Ma, may have resulted from subsequent population expansions across cyclic interglacial periods, during which wider suitable areas were available for colonization. Because the probability of coalescence is inversely related to the number of gene copies (Kingman, 1982), most coalescence would occur just before demographic expansion, when populations had smaller effective population sizes (see (Excoffier et al., 2009) for a review). After diversification, some lineages became widespread through dispersal during interglacial periods (Figure 6), most likely due to range expansion.

Lineage dispersal was mainly from three populations: CAC, SCA, FAT, and PTU (see Figure 5). This diffusion pattern explains the widespread geographical distribution of some haplotypes (e.g., H1 and H14, see Figures 1, 3) and the restricted geographical distribution of other haplotypes that diverged more recently (e.g., H2, H17, H18, see Figures 1, 3), leading to high population differentiation. Our results also evidenced *in situ* diversification of many haplotypes in SAF (see Figure 5) with restricted geographical distribution. This population is at the edge of the *T. aurea* historical refugium and is now an isolated savanna remaining in the transition between savanna and caatinga woodlands (Caatinga biome). Although our findings show possible connections among SAF and other populations (wide historical refugium and dispersal of H25 from SCA to SAF with posterior extinction), we hypothesize that the relative isolation may have precluded the dispersal of lineages from SAF to other populations. It is important to note that events of long distance dispersal, such as from SCA to SAF, are most likely due to stepping-stone dispersal and would be detected only with continuous spatial sampling. However, continuously sampling in large scale is hindered by the high savanna fragmentation in Brazil. Besides, estimates of the mean rate of dispersal equal to 1.17 km/yr using RRW is congruent with the pollen dispersal distance reported for *T. aurea* (~2.0 km, Braga and Collevatti, 2011). Gene dispersal estimate (~300 m, Braga and Collevatti, 2011) based on spatial genetic structure was lower than pollen dispersal. Although this method may underestimate seed dispersal distance, long-distance dispersal is mainly due to pollen dispersal, which may also explain widespread distribution of ITS haplotypes (see also the discussion below).

The consistence among predictions is still evidence of genetic connectivity. Despite the range and demographical expansions through the time, ENMs suggested that all populations, except for SUM and SCA, are inside the historical refugium; i.e., areas with high climatic suitability throughout time (Figure 7D). Such evidence suggests that even with the smaller geographical range during LGM and spatial displacement during warming phases, a wide climatically stable area was still available throughout the last glacial cycle, favoring population persistence and genetic connectivity. If this stable area was maintained during the recurrent glaciations of the Quaternary, we can predict long-term genetic connectivity among *T. aurea* populations, which is still supported by a high density of well-supported links among populations inside that historical refugium (Figure 6). Genetic connectivity may have also been maintained by the long-distance pollen and seed dispersal of *T. aurea* (Braga and Collevatti, 2011). Thus, the low haplotype diversity within a population may be an effect of selective sweeps. For instance, Fu's neutrality test was significant, and negative *FS* values potentially indicate selective sweeps of chloroplast genomes, as reported for other plant species (e.g., Kapralov and Filatov, 2007). In addition, despite differences in effective population size and inheritance, chloroplast genome showed higher diversity than ITS nrDNA, that has four times the effective size of chloroplast genome. This may be due to concerted evolution in nrDNA that may decrease genetic variation (Ohta, 1984). Complementarily, the cycles of range retraction and expansion during recurrent glaciations, may have caused the extinction of haplotypes in some populations and a spatial assortment decreasing genetic diversity, as suggested by similar distribution dynamics along correspondent glacial and interglacial phases from older glacial cycles, (see Hewitt, 1996; Excoffier and Ray, 2008; Arenas et al., 2012). For instance, the findings from RRW showed that the chloroplast haplotype H14 (ITS H1) dispersed from population PTU to SDO (~1250–900 ka, see Figure 6) and became extinct in SDO after dispersion to VIB and STZ (~1050–900 ka). Extinction of this haplotype may also have occurred in SCA, after dispersion to PTU and PAN (~1350 ka). In addition, recent anthropogenic disturbs may also have caused bottlenecks, decreasing genetic diversity in *T. aurea* populations because some populations, such as BAR, STZ, ARA, which are isolated remnant individuals in pasture or crop plantations have low genetic diversity. We believe that our results are not an artifact of sampling effort because populations with larger sample sizes had the same number of haplotypes as populations with smaller sample sizes.

Actually, the pattern of lineage spread and extinction may unravel the low genetic diversity in many populations, even in the center of species geographical range. In fact, populations in more instable areas in eastern and western edges of the historical refugium (CAC, CHG, PTU, SCA) have high genetic diversity. We hypothesize that these populations, at the edge of historical refugium may represent “stable rear edges,” i.e., relict populations that have persisted in suitable local habitats across the Quaternary glaciation cycles (see Petit et al., 2003; Hampe and Petit, 2005). Moreover, along with high genetic diversity, CAC, CHG, PTU, and SCA also present the oldest lineages sampled in this study (see Figures 4, 6).

Finally, the scenario of range and demographic expansion through time for *T. aurea* was also supported for other Brazilian savanna species such as *Caryocar brasiliense* (Collevatti et al., 2012b) and *Dipteryx alata* (Collevatti et al., 2013b) and for a Brazilian savanna swamp palm species, *Mauritia flexuosa* (Lima et al., 2014). The few phylogeographical studies performed so far with savanna species (e.g., Ramos et al., 2007; Novaes et al., 2010, 2013; Collevatti et al., 2012b) have also shown high levels of genetic differentiation among populations and evidences of colonization of southeastern Brazilian savannas during warming interglacial periods of the Quaternary.

In conclusion, our findings suggest that the pattern of genetic diversity in *T. aurea* may be the outcome of population expansion through warming periods of the Quaternary, with recurrent events of spatial displacements along the cyclic events of glaciations. We are aware of the differences in the time scale of predictions from different approaches, given that the RRW predicts spatial displacements occurring in a deeper time than ENMs. However, all approaches converge to consistent patterns: general dynamics of *T. aurea* following suitable climatic conditions toward the northern regions during warming periods in the early Quaternary, with retraction of populations during glacial phases, and new dispersal events at the middle and end of the Quaternary. Such consistent patterns from different methods and analyses indicate that our multi-model inference approach is dynamic and flexible, allowing the inclusion of new components to directly infer processes affecting phylogeographic patterns through time, and promises advances in hypothesis testing in phylogeography.

## Author contributions

RC and ML conceived the overall study. SR generated the genetic data. RC, LT, and ML analyzed the data and wrote the manuscript. All authors approved the final version of the manuscript.

### Conflict of interest statement

The authors declare that the research was conducted in the absence of any commercial or financial relationships that could be construed as a potential conflict of interest.
