# Supplementary material for: Relaxed random walk model coupled with ecological niche modeling unravel the dispersal dynamics of a Neotropical savanna tree species in the deeper Quaternary
Source: Front Plant Sci. 2015 Aug 25;6:653. doi: 10.3389/fpls.2015.00653 (PMC4548090; doi:10.3389/fpls.2015.00653)
Supplement: Supplementary file 1 [file Table1.DOCX]

Frontiers in Plant Science

Supporting Information

**Relaxed random walk model coupled with ecological niche modeling unravel the dispersal dynamics of a Neotropical savanna tree species in the deeper Quaternary**

Rosane G. Collevatti, Levi Carina Terribile, Suelen G. Rabelo, Matheus S. Lima-Ribeiro

**Appendix S1 Tables**

**Table S1** Sampling location for the 21 populations of *Tabebuia aurea* from Brazil used for genetic analyses and species sampled as outgroups.

| Code | Population Locality | Latitude | Longitude |
| --- | --- | --- | --- |
| **AGE** | Águas Emendadas Ecological Reserve, DF | S15 35 04.7 | W47 39 59.7 |
| **ARA** | Formoso do Araguaia, TO | S11 33 43.9 | W49 48 15.9 |
| **BAG** | Barra do Garça, MT | S15 00 47.3 | W51 16 41.7 |
| **BAR** | Barreiras, BA | S12 05 06.3 | W45 26 50.8 |
| **BOD** | Aquidauana, MS | S20 35 59.2 | W56 00 12.4 |
| **CAC** | Cáceres, MT | S16 08 23.7 | W57 59 19.8 |
| **CHG** | Chapada dos Guimarães, MT | S15 23 45.0 | W55 50 00.7 |
| **FAT** | Fátima, TO | S10 48 20.8 | W48 54 22.9 |
| **GSV** | Chapada Gaúcha, MG | S15 13 25.6 | W45 49 12.0 |
| **NIQ** | Niquelândia, GO | S14 11 39.7 | W48 18 38.2 |
| **PAN** | Januária, MG | S15 30 53.6 | W44 41 32.5 |
| **PNE** | Mineiros, GO | S17 54 43.2 | W52 21 08.9 |
| **POT** | Portelândia, GO | S17 19 31.6 | W52 42 17.1 |
| **PTU** | Paracatu, MG | S17 27 44.3 | W46 39 26.5 |
| **SAF** | Santa Filomena, PI | S9 10 34.5 | W45 32 25.0 |
| **SCA** | Serra da Canastra, MG | S19 34 25.6 | W46 29 56.1 |
| **SDO** | Serra Dourada, GO | S16 04 02.1 | W50 10 35.6 |
| **SEC** | Campo Maior, PI | S4 51 37.4 | W42 03 36.5 |
| **STZ** | Santa Terezinha de Goiás, GO | S14 21 07.0 | W49 30 15.8 |
| **SUM** | Lagoa Santa, MG | S19 32 24.3 | W43 56 02.2 |
| **VIB** | Vila Boa, GO | S15 00 46.0 | W47 02 32.9 |
| **TCH** | Sapucaia, RJ | S21 58 28.7 | W42 55 26.6 |
| **TIM** | Altamiro Pacheco, GO | S16 32 20.1 | W49 08 17.5 |
| **CYA** | Sapucaia, RJ | S21 58 28.7 | W42 55 26.6 |

**Table S2** Contemporary occurrence records (237) of *Tabebuia aurea* represented by the centroid of grid cells across the Neotropics used in the ecological niche modelling (ENM)**.**

| **Long** | **Lat** |  | **Long** | **Lat** |  | **Long** | **Lat** |  | **Long** | **Lat** |
| --- | --- | --- | --- | --- | --- | --- | --- | --- | --- | --- |
| -62.25 | -14.25 |  | -51.25 | -22.25 |  | -47.75 | -18.25 |  | -42.25 | -13.75 |
| -61.75 | -14.75 |  | -51.25 | -18.75 |  | -47.75 | -16.75 |  | -42.25 | -13.25 |
| -61.25 | -15.25 |  | -51.25 | -18.25 |  | -47.75 | -16.25 |  | -42.25 | -9.75 |
| -61.25 | -14.75 |  | -51.25 | -16.75 |  | -47.75 | -15.75 |  | -41.75 | -13.25 |
| -60.75 | -14.75 |  | -51.25 | -14.75 |  | -47.75 | -15.25 |  | -41.75 | -4.75 |
| -60.75 | -13.75 |  | -50.75 | -22.25 |  | -47.75 | -9.25 |  | -41.75 | -4.25 |
| -60.25 | -17.75 |  | -50.75 | -17.75 |  | -47.75 | -6.25 |  | -41.25 | -16.25 |
| -60.25 | -15.75 |  | -50.75 | -14.25 |  | -47.25 | -22.25 |  | -41.25 | -12.75 |
| -59.75 | -18.25 |  | -50.25 | -24.75 |  | -47.25 | -20.25 |  | -41.25 | -8.25 |
| -59.25 | -19.25 |  | -50.25 | -22.75 |  | -47.25 | -19.25 |  | -40.75 | -9.75 |
| -59.25 | -18.25 |  | -50.25 | -20.75 |  | -47.25 | -15.75 |  | -40.25 | -9.25 |
| -59.25 | -14.25 |  | -50.25 | -16.75 |  | -47.25 | -15.25 |  | -40.25 | -7.75 |
| -58.75 | -17.75 |  | -50.25 | -16.25 |  | -47.25 | -13.75 |  | -39.75 | -10.75 |
| -58.25 | -19.75 |  | -50.25 | -15.75 |  | -47.25 | -6.75 |  | -39.75 | -9.25 |
| -58.25 | -16.75 |  | -50.25 | -13.25 |  | -47.25 | -6.25 |  | -39.75 | -8.75 |
| -58.25 | -16.25 |  | -49.75 | -20.75 |  | -46.75 | -22.25 |  | -39.25 | -16.25 |
| -57.75 | -19.25 |  | -49.75 | -17.25 |  | -46.75 | -18.75 |  | -39.25 | -12.75 |
| -57.75 | -17.75 |  | -49.75 | -10.75 |  | -46.75 | -17.75 |  | -39.25 | -10.25 |
| -57.75 | -16.25 |  | -49.25 | -24.25 |  | -46.75 | -17.25 |  | -39.25 | -9.75 |
| -57.25 | -22.75 |  | -49.25 | -22.25 |  | -46.75 | -14.25 |  | -39.25 | -9.25 |
| -57.25 | -22.25 |  | -49.25 | -19.25 |  | -46.75 | -13.75 |  | -39.25 | -8.75 |
| -57.25 | -21.75 |  | -49.25 | -16.75 |  | -46.75 | -10.75 |  | -39.25 | -7.25 |
| -57.25 | -19.75 |  | -49.25 | -16.25 |  | -46.25 | -18.75 |  | -39.25 | -5.25 |
| -57.25 | -17.75 |  | -49.25 | -15.75 |  | -46.25 | -16.25 |  | -38.75 | -9.75 |
| -56.75 | -20.75 |  | -49.25 | -15.25 |  | -46.25 | -13.25 |  | -38.75 | -8.75 |
| -56.75 | -19.75 |  | -49.25 | -14.25 |  | -45.75 | -16.25 |  | -38.75 | -4.25 |
| -56.75 | -19.25 |  | -49.25 | -13.75 |  | -45.75 | -15.25 |  | -38.75 | -3.75 |
| -56.75 | -18.75 |  | -49.25 | -12.75 |  | -45.75 | -12.25 |  | -38.25 | -11.25 |
| -56.75 | -16.75 |  | -49.25 | -12.25 |  | -45.75 | -11.75 |  | -38.25 | -10.25 |
| -56.75 | -16.25 |  | -49.25 | -11.75 |  | -45.25 | -16.75 |  | -38.25 | -9.75 |
| -56.25 | -20.25 |  | -48.75 | -23.25 |  | -45.25 | -16.25 |  | -38.25 | -9.25 |
| -56.25 | -15.75 |  | -48.75 | -22.75 |  | -45.25 | -12.75 |  | -38.25 | -8.75 |
| -56.25 | -14.25 |  | -48.75 | -22.25 |  | -45.25 | -12.25 |  | -38.25 | -6.75 |
| -55.75 | -22.25 |  | -48.75 | -20.75 |  | -45.25 | -11.25 |  | -38.25 | -6.25 |
| -55.75 | -21.25 |  | -48.75 | -20.25 |  | -45.25 | -10.25 |  | -37.75 | -9.75 |
| -55.75 | -20.25 |  | -48.75 | -19.25 |  | -45.25 | -9.75 |  | -37.75 | -9.25 |
| -55.75 | -16.25 |  | -48.75 | -18.75 |  | -45.25 | -7.25 |  | -37.75 | -8.25 |
| -55.75 | -15.25 |  | -48.75 | -17.75 |  | -44.75 | -14.25 |  | -37.25 | -10.75 |
| -55.75 | -14.75 |  | -48.75 | -16.25 |  | -44.75 | -13.25 |  | -37.25 | -10.25 |
| -55.75 | -9.75 |  | -48.75 | -15.75 |  | -44.75 | -12.25 |  | -37.25 | -9.75 |
| -55.25 | -21.75 |  | -48.75 | -13.75 |  | -44.75 | -11.75 |  | -37.25 | -8.25 |
| -55.25 | -20.25 |  | -48.75 | -8.75 |  | -44.75 | -11.25 |  | -37.25 | -7.25 |
| -54.75 | -22.75 |  | -48.75 | -8.25 |  | -44.75 | -10.75 |  | -37.25 | -6.75 |
| -54.75 | -22.25 |  | -48.75 | -0.75 |  | -44.25 | -20.75 |  | -36.75 | -10.25 |
| -54.75 | -20.25 |  | -48.25 | -22.75 |  | -44.25 | -19.25 |  | -36.75 | -9.75 |
| -54.75 | -14.75 |  | -48.25 | -18.75 |  | -44.25 | -15.25 |  | -36.75 | -8.75 |
| -54.75 | -2.75 |  | -48.25 | -18.25 |  | -44.25 | -13.75 |  | -36.75 | -7.25 |
| -54.75 | -1.75 |  | -48.25 | -15.75 |  | -44.25 | -4.25 |  | -36.75 | -6.75 |
| -53.25 | -22.25 |  | -48.25 | -14.25 |  | -44.25 | -3.75 |  | -36.75 | -6.25 |
| -52.75 | -16.25 |  | -48.25 | -13.75 |  | -44.25 | -2.75 |  | -36.25 | -9.25 |
| -52.25 | -21.75 |  | -48.25 | -13.25 |  | -43.75 | -19.75 |  | -36.25 | -8.25 |
| -52.25 | -15.75 |  | -48.25 | -10.25 |  | -43.75 | -18.75 |  | -36.25 | -7.75 |
| -52.25 | -14.75 |  | -48.25 | -8.25 |  | -43.75 | -18.25 |  | -36.25 | -7.25 |
| -52.25 | -13.75 |  | -48.25 | -7.25 |  | -43.75 | -15.25 |  | -35.75 | -8.25 |
| -51.75 | -20.75 |  | -48.25 | -5.75 |  | -43.75 | -6.75 |  | -35.75 | -6.75 |
| -51.75 | -18.25 |  | -47.75 | -22.75 |  | -43.25 | -9.25 |  | -35.25 | -7.75 |
| -51.75 | -17.75 |  | -47.75 | -22.25 |  | -43.25 | -4.75 |  | -35.25 | -5.75 |
| -51.75 | -17.25 |  | -47.75 | -21.25 |  | -42.75 | -10.75 |  |  |  |
| -51.75 | -16.75 |  | -47.75 | -19.75 |  | -42.75 | -5.25 |  |  |  |
| -51.75 | -12.75 |  | -47.75 | -19.25 |  | -42.75 | -3.75 |  |  |  |

**Table S3** Details on the paleoclimatic simulations (AOGCMs) used in the ecological niche modeling of *Tabebuia aurea.*

| **Model ID** | **Modeling Center** | **Resolution*** | **Source** | **Year** |
| --- | --- | --- | --- | --- |
| CCSM4 | University of Miami – RSMAS. USA | 0.9° × 1.25° | CMIP5/PMIP3 | 2012 |
| CNRM-CM5 | Centre National de Recherches Meteorologiques / Centre Europeen de Recherche et Formation Avancees en Calcul Scientifique. France | 1.4° x 1.4° | CMIP5/PMIP3 | 2012 |
| MIROC-ESM | Atmosphere and Ocean Research Institute (University of Tokyo). National Institute for Environmental Studies. and Japan Agency for Marine-Earth Science and Technology. Japan | 2.8° × 2.8° | CMIP5/PMIP3 | 2012 |
| MRI-CGCM3 | Meteorological Research Institute. Japan | 1.1° x 1.1° | CMIP5/PMIP3 | 2012 |

* longitude × latitude

CMIP5 – Coupled Model Intercomparison Project. Phase 5 (<http://cmip-pcmdi.llnl.gov/>)

PMIP3 – Paleoclimate Modelling Intercomparison Project. Phase 3 (<http://pmip3.lsce.ipsl.fr/>)

**Table S4** Ecological niche modeling methods used to estimate *Tabebuia aurea* potential distribution.

| **Method** | **Species data type** |
| --- | --- |
| Bioclimatic Envelope (BIOCLIM) | Presence only |
| Ecological Niche Factor Analysis (ENFA) | Presence only |
| Euclidian Distance (EuclidDist) | Presence only |
| Generalized Linear Models (GLM) | Presence and absence |
| Gower Distance (GowerDist) | Presence only |
| Mahalanobis Distance (MahalDist) | Presence only |
| Maximum Entropy (Maxent) | Presence/background |
| Generalized additive models (GAM) | Presence and absence |
| Flexible discriminant analysis (FDA) | Presence and absence |
| Multivariate adaptive regression splines (MARS) | Presence and absence |
| Generalized boosted models (GBM) | Presence and absence |
| Neural Networks (ANN) | Presence and absence |
| Random Forest (RNDFOR) | Presence and absence |

**Table S5** Values of True Skill Statistics (TSS) with mean and standard deviation for all ENM*AOGCM’s combinations from ecological niche modeling of *Tabebuia aurea*.

|  |  | **AOGCMs** | | | | | | |
| --- | --- | --- | --- | --- | --- | --- | --- | --- |
|  |  | | CCSM | CNRM | MIROC | MRI | **Mean** | **SD** |
| **ENM Algorithms** | **BioClim** | | 0.5 | 0.6 | 0.5 | 0.5 | 0.5 | 0.1 |
|  | **ENFA** | | 0.4 | 0.4 | 0.4 | 0.3 | 0.4 | 0.0 |
|  | **EuclidDist** | | 0.5 | 0.5 | 0.4 | 0.5 | 0.5 | 0.1 |
|  | **FDA** | | 0.6 | 0.6 | 0.6 | 0.6 | 0.6 | 0.0 |
|  | **GAM** | | 0.7 | 0.7 | 0.6 | 0.6 | 0.6 | 0.0 |
|  | **GBM** | | 0.7 | 0.6 | 0.6 | 0.6 | 0.6 | 0.1 |
|  | **GLM** | | 0.6 | 0.6 | 0.6 | 0.6 | 0.6 | 0.0 |
|  | **GowerDist** | | 0.5 | 0.6 | 0.5 | 0.5 | 0.5 | 0.1 |
|  | **MahalanobisDist** | | 0.5 | 0.6 | 0.5 | 0.5 | 0.5 | 0.1 |
|  | **MARS** | | 0.6 | 0.6 | 0.5 | 0.6 | 0.6 | 0.0 |
|  | **MaxEnt** | | 0.6 | 0.6 | 0.6 | 0.6 | 0.6 | 0.0 |
|  | **NNet** | | 0.6 | 0.6 | 0.6 | 0.6 | 0.6 | 0.0 |
|  | **RndFor** | | 0.7 | 0.7 | 0.6 | 0.6 | 0.7 | 0.0 |
|  | **Mean** | | 0.6 | 0.6 | 0.5 | 0.5 | *0.6* | *0.0* |
|  | **SD** | | 0.1 | 0.1 | 0.1 | 0.1 | *0.1* |  |

**Table S6** Pairwise *F_ST_* among the 21 populations of *Tabebuia aurea* sampled in Brazil. Chloroplast below diagonal and nuclear ITS above diagonal. All *F_ST_* values for chloroplast are significant and values in bold are significant for nuclear ITS, *p* < 0.05.

| **Pop** | **AGE** | **ARA** | **BAG** | **BAR** | **BOD** | **CAC** | **CHG** | **FAT** | **GSV** | **NIQ** | **PAN** | **PNE** | **POT** | **PTU** | **SAF** | **SCA** | **SDO** | **SEC** | **SUM** | **STZ** | **VIB** |
| --- | --- | --- | --- | --- | --- | --- | --- | --- | --- | --- | --- | --- | --- | --- | --- | --- | --- | --- | --- | --- | --- |
| **AGE** | 0.000 | 0.000 | 0.000 | 0.000 | 0.000 | 0.000 | **0.999** | 0.066 | 0.000 | 0.000 | 0.000 | 0.000 | 0.000 | 0.000 | 0.000 | 0.000 | 0.000 | **0.745** | 0.000 | 0.052 | 0.000 |
| **ARA** | 0.117 | 0.000 | 0.000 | 0.000 | 0.000 | 0.000 | **0.999** | 0.056 | 0.000 | 0.000 | 0.000 | 0.000 | 0.000 | 0.000 | 0.000 | 0.000 | 0.000 | **0.745** | 0.000 | 0.052 | 0.000 |
| **BAG** | 0.035 | 0.990 | 0.000 | 0.000 | 0.000 | 0.000 | **0.999** | 0.075 | 0.000 | 0.000 | 0.000 | 0.000 | 0.000 | 0.000 | 0.000 | 0.000 | 0.000 | **0.714** | 0.000 | 0.034 | 0.000 |
| **BAR** | 0.121 | 0.995 | 1.000 | 0.000 | 0.000 | 0.000 | **0.998** | 0.044 | 0.000 | 0.000 | 0.000 | 0.000 | 0.000 | 0.000 | 0.000 | 0.000 | 0.000 | **0.651** | 0.000 | 0.000 | 0.000 |
| **BOD** | 0.031 | 0.990 | 0.000 | 1.000 | 0.000 | 0.000 | **0.999** | 0.070 | 0.000 | 0.000 | 0.000 | 0.000 | 0.000 | 0.000 | 0.000 | 0.000 | 0.000 | **0.714** | 0.000 | 0.034 | 0.000 |
| **CAC** | 0.273 | 0.401 | 0.423 | 0.353 | 0.414 | 0.000 | **0.999** | 0.065 | 0.000 | 0.000 | 0.000 | 0.000 | 0.000 | 0.000 | 0.000 | 0.000 | 0.000 | **0.651** | 0.000 | 0.000 | 0.000 |
| **CHG** | 0.771 | 0.892 | 0.904 | 0.882 | 0.902 | 0.351 | 0.000 | **0.925** | **0.999** | **0.999** | **0.999** | **0.999** | **0.999** | **0.999** | **0.999** | **0.999** | **0.998** | **0.995** | **0.999** | **0.997** | **0.999** |
| **FAT** | 0.201 | 0.748 | 0.656 | 0.552 | 0.689 | 0.444 | 0.901 | 0.000 | **0.097** | 0.058 | 0.005 | 0.009 | 0.009 | 0.009 | 0.050 | 0.050 | **0.999** | 0.000 | 0.012 | 0.031 | 0.012 |
| **GSV** | 0.148 | 0.971 | 0.973 | 0.925 | 0.972 | 0.314 | 0.861 | 0.376 | 0.000 | 0.000 | 0.000 | 0.000 | 0.000 | 0.000 | 0.000 | 0.000 | 0.000 | **0.496** | 0.000 | 0.078 | 0.000 |
| **NIQ** | 0.136 | 0.859 | 0.824 | 0.788 | 0.819 | 0.384 | 0.886 | 0.363 | 0.348 | 0.000 | 0.000 | 0.000 | 0.000 | 0.000 | 0.000 | 0.000 | 0.000 | **0.588** | 0.000 | 0.031 | 0.000 |
| **PAN** | 0.195 | 0.921 | 0.908 | 0.824 | 0.906 | 0.405 | 0.894 | 0.377 | 0.068 | 0.140 | 0.000 | 0.000 | 0.000 | 0.000 | 0.000 | 0.000 | 0.000 | **0.758** | 0.000 | 0.060 | 0.000 |
| **PNE** | 0.118 | 0.957 | 0.943 | 0.968 | 0.941 | 0.417 | 0.898 | 0.603 | 0.889 | 0.438 | 0.755 | 0.000 | 0.000 | 0.000 | 0.000 | 0.000 | 0.000 | **0.769** | 0.000 | 0.068 | 0.000 |
| **POT** | 0.129 | 0.994 | 1.000 | 1.000 | 1.000 | 0.419 | 0.899 | 0.618 | 0.947 | 0.483 | 0.800 | 0.067 | 0.000 | 0.000 | 0.000 | 0.000 | 0.000 | **0.769** | 0.000 | 0.068 | 0.000 |
| **PTU** | 0.242 | 0.545 | 0.546 | 0.545 | 0.537 | 0.383 | 0.832 | 0.533 | 0.443 | 0.452 | 0.506 | 0.467 | 0.469 | 0.000 | 0.000 | 0.000 | 0.000 | **0.496** | 0.000 | 0.078 | 0.000 |
| **SAF** | 0.766 | 0.945 | 0.951 | 0.942 | 0.949 | 0.684 | 0.909 | 0.937 | 0.928 | 0.935 | 0.942 | 0.944 | 0.946 | 0.789 | 0.000 | 0.000 | 0.000 | **0.588** | 0.000 | 0.031 | 0.000 |
| **SCA** | 0.214 | 0.590 | 0.577 | 0.597 | 0.569 | 0.386 | 0.855 | 0.544 | 0.486 | 0.436 | 0.535 | 0.477 | 0.483 | 0.255 | 0.863 | 0.000 | 0.000 | **0.588** | 0.000 | 0.031 | 0.000 |
| **SDO** | 0.127 | 0.997 | 1.000 | 1.000 | 1.000 | 0.215 | 0.826 | 0.673 | 0.924 | 0.800 | 0.855 | 0.968 | 1.000 | 0.406 | 0.912 | 0.203 | 0.000 | 0**.333** | 0.000 | 0.001 | 0.000 |
| **SEC** | 0.172 | 0.996 | 1.000 | 1.000 | 1.000 | 0.255 | 0.836 | 0.687 | 0.932 | 0.812 | 0.863 | 0.970 | 1.000 | 0.437 | 0.917 | 0.245 | 0.000 | 0.000 | **0.514** | **0.714** | **0.714** |
| **SUM** | 0.326 | 0.991 | 0.993 | 0.988 | 0.993 | 0.413 | 0.892 | 0.771 | 0.949 | 0.875 | 0.904 | 0.972 | 0.991 | 0.585 | 0.944 | 0.463 | 0.897 | 1.000 | 0.000 | 0.000 | 0.000 |
| **STZ** | 0.332 | 0.998 | 1.000 | 1.000 | 1.000 | 0.439 | 0.902 | 0.802 | 0.974 | 0.903 | 0.928 | 0.984 | 1.000 | 0.619 | 0.951 | 0.514 | 1.000 | 0.947 | 0.903 | 0.000 | 0.034 |
| **VIB** | 0.347 | 0.995 | 0.998 | 0.996 | 0.997 | 0.434 | 0.899 | 0.796 | 0.967 | 0.896 | 0.922 | 0.980 | 0.997 | 0.610 | 0.949 | 0.504 | 0.973 | 0.983 | 0.975 | 0.888 | 0.000 |

**Table S7** Number of migrants per generation (*N_e_m* ) for the 21 populations of *Tabebuia aurea* in Brazil, based on Bayesian coalescent analysis. Migration direction is from populations in the columns into populations in the rows. Note that all values of *N_e_m* are < 1.00.

| **Pop** | **AGE** | **ARA** | **BAG** | **BAR** | **BOD** | **CAR** | **CHG** | **FAT** | **GSV** | **NIQ** | **PAN** | **PNE** | **POT** | **PTU** | **SAF** | **SCA** | **SDO** | **SEC** | **STZ** | **SUM** | **VIB** |
| --- | --- | --- | --- | --- | --- | --- | --- | --- | --- | --- | --- | --- | --- | --- | --- | --- | --- | --- | --- | --- | --- |
| **AGE** |  | 0.008 | 0.006 | 0.003 | 0.002 | 0.113 | 0.221 | 0.098 | 0.046 | 0.002 | 0.006 | 0.017 | 0.002 | 0.139 | 0.423 | 0.270 | 0.006 | 0.025 | 0.050 | 0.015 | 0.008 |
| **ARA** | 0.047 |  | 0.005 | 0.003 | 0.002 | 0.154 | 0.230 | 0.112 | 0.028 | 0.003 | 0.005 | 0.011 | 0.002 | 0.093 | 0.386 | 0.225 | 0.007 | 0.016 | 0.048 | 0.012 | 0.012 |
| **BAG** | 0.068 | 0.007 |  | 0.003 | 0.002 | 0.125 | 0.145 | 0.129 | 0.028 | 0.004 | 0.005 | 0.015 | 0.002 | 0.098 | 0.750 | 0.205 | 0.005 | 0.022 | 0.045 | 0.010 | 0.007 |
| **BAR** | 0.048 | 0.008 | 0.005 |  | 0.002 | 0.126 | 0.128 | 0.112 | 0.028 | 0.003 | 0.005 | 0.018 | 0.002 | 0.120 | 0.367 | 0.270 | 0.005 | 0.016 | 0.049 | 0.011 | 0.007 |
| **BOD** | 0.072 | 0.011 | 0.005 | 0.003 |  | 0.123 | 0.255 | 0.120 | 0.027 | 0.002 | 0.007 | 0.011 | 0.002 | 0.104 | 0.394 | 0.177 | 0.006 | 0.025 | 0.046 | 0.010 | 0.010 |
| **CAR** | 0.046 | 0.008 | 0.007 | 0.003 | 0.002 |  | 0.131 | 0.158 | 0.028 | 0.002 | 0.005 | 0.020 | 0.002 | 0.103 | 0.774 | 0.209 | 0.005 | 0.019 | 0.045 | 0.010 | 0.008 |
| **CHG** | 0.068 | 0.014 | 0.007 | 0.003 | 0.003 | 0.119 |  | 0.117 | 0.029 | 0.002 | 0.005 | 0.013 | 0.003 | 0.106 | 0.637 | 0.329 | 0.007 | 0.019 | 0.049 | 0.013 | 0.008 |
| **FAT** | 0.069 | 0.010 | 0.005 | 0.003 | 0.002 | 0.145 | 0.140 |  | 0.041 | 0.003 | 0.005 | 0.011 | 0.002 | 0.115 | 0.357 | 0.203 | 0.005 | 0.016 | 0.048 | 0.011 | 0.008 |
| **GSV** | 0.074 | 0.008 | 0.005 | 0.003 | 0.003 | 0.110 | 0.193 | 0.098 |  | 0.002 | 0.005 | 0.013 | 0.002 | 0.124 | 0.597 | 0.173 | 0.005 | 0.017 | 0.047 | 0.015 | 0.007 |
| **NIQ** | 0.072 | 0.008 | 0.005 | 0.003 | 0.002 | 0.110 | 0.138 | 0.155 | 0.033 |  | 0.007 | 0.014 | 0.003 | 0.095 | 0.370 | 0.237 | 0.006 | 0.018 | 0.060 | 0.011 | 0.010 |
| **PAN** | 0.047 | 0.008 | 0.005 | 0.003 | 0.003 | 0.127 | 0.196 | 0.108 | 0.036 | 0.003 |  | 0.012 | 0.002 | 0.130 | 0.370 | 0.239 | 0.005 | 0.016 | 0.078 | 0.016 | 0.008 |
| **PNE** | 0.073 | 0.009 | 0.005 | 0.003 | 0.003 | 0.150 | 0.137 | 0.094 | 0.035 | 0.003 | 0.006 |  | 0.003 | 0.103 | 0.864 | 0.184 | 0.006 | 0.025 | 0.049 | 0.010 | 0.008 |
| **POT** | 0.047 | 0.012 | 0.005 | 0.003 | 0.002 | 0.104 | 0.230 | 0.124 | 0.029 | 0.003 | 0.007 | 0.014 |  | 0.099 | 0.602 | 0.186 | 0.005 | 0.016 | 0.079 | 0.010 | 0.008 |
| **PTU** | 0.071 | 0.008 | 0.005 | 0.003 | 0.002 | 0.117 | 0.157 | 0.095 | 0.033 | 0.002 | 0.005 | 0.011 | 0.002 |  | 0.769 | 0.295 | 0.005 | 0.016 | 0.050 | 0.010 | 0.008 |
| **SAF** | 0.080 | 0.008 | 0.005 | 0.003 | 0.003 | 0.180 | 0.289 | 0.098 | 0.029 | 0.002 | 0.007 | 0.015 | 0.002 | 0.102 |  | 0.179 | 0.005 | 0.016 | 0.046 | 0.010 | 0.008 |
| **SCA** | 0.046 | 0.009 | 0.005 | 0.004 | 0.002 | 0.108 | 0.236 | 0.118 | 0.026 | 0.002 | 0.005 | 0.011 | 0.002 | 0.144 | 0.383 |  | 0.007 | 0.021 | 0.050 | 0.012 | 0.013 |
| **SDO** | 0.049 | 0.009 | 0.005 | 0.003 | 0.002 | 0.105 | 0.138 | 0.104 | 0.030 | 0.002 | 0.005 | 0.011 | 0.003 | 0.135 | 0.381 | 0.280 |  | 0.026 | 0.053 | 0.011 | 0.010 |
| **SEC** | 0.049 | 0.009 | 0.007 | 0.004 | 0.003 | 0.104 | 0.241 | 0.094 | 0.028 | 0.003 | 0.004 | 0.011 | 0.002 | 0.102 | 0.334 | 0.235 | 0.005 |  | 0.076 | 0.010 | 0.008 |
| **STZ** | 0.074 | 0.008 | 0.007 | 0.003 | 0.003 | 0.156 | 0.135 | 0.152 | 0.031 | 0.003 | 0.005 | 0.012 | 0.002 | 0.140 | 0.414 | 0.189 | 0.006 | 0.015 |  | 0.010 | 0.008 |
| **SUM** | 0.049 | 0.008 | 0.006 | 0.004 | 0.003 | 0.112 | 0.146 | 0.158 | 0.028 | 0.002 | 0.005 | 0.011 | 0.002 | 0.097 | 0.606 | 0.209 | 0.005 | 0.016 | 0.047 |  | 0.009 |
| **VIB** | 0.048 | 0.008 | 0.006 | 0.003 | 0.002 | 0.154 | 0.158 | 0.128 | 0.028 | 0.002 | 0.005 | 0.014 | 0.002 | 0.091 | 0.556 | 0.214 | 0.005 | 0.016 | 0.098 | 0.013 |  |

**Table S8** Credibility interval (95%) of the number of migrants per generation (see Table S7 for the number of migrants) for the 21 populations of *Tabebuia aurea* in Brazil, based on Bayesian coalescent analysis. Credibility interval for migration is from populations in the columns into populations in the rows. All values are > 0.000 (approximate decimal places).

|  | **AGE** | | **ARA** | | **BAG** | | **BAR** | | **BOD** | | **CAR** | | **CHG** | | **FAT** | | **GSV** | | **NIQ** | | **PAN** | | **PNE** | | **POT** | | **PTU** | | **SAF** | | **SCA** | | **SDO** | | **SEC** | | **STZ** | | **SUM** | | **VIB** | |
| --- | --- | --- | --- | --- | --- | --- | --- | --- | --- | --- | --- | --- | --- | --- | --- | --- | --- | --- | --- | --- | --- | --- | --- | --- | --- | --- | --- | --- | --- | --- | --- | --- | --- | --- | --- | --- | --- | --- | --- | --- | --- | --- |
| **AGE** |  |  | 0.000 | 0.034 | 0.000 | 0.058 | 0.000 | 0.013 | 0.000 | 0.010 | 0.000 | 0.583 | 0.011 | 0.732 | 0.000 | 0.462 | 0.003 | 0.166 | 0.000 | 0.025 | 0.000 | 0.028 | 0.001 | 0.059 | 0.000 | 0.009 | 0.017 | 0.488 | 0.001 | 2.113 | 0.032 | 0.874 | 0.000 | 0.027 | 0.000 | 0.209 | 0.000 | 0.198 | 0.001 | 0.054 | 0.000 | 0.046 |
| **ARA** | 0.000 | 0.196 |  |  | 0.000 | 0.054 | 0.000 | 0.012 | 0.000 | 0.010 | 0.010 | 0.606 | 0.013 | 0.732 | 0.000 | 0.463 | 0.000 | 0.157 | 0.000 | 0.026 | 0.000 | 0.026 | 0.000 | 0.055 | 0.000 | 0.009 | 0.001 | 0.450 | 0.000 | 2.106 | 0.003 | 0.843 | 0.000 | 0.029 | 0.000 | 0.188 | 0.000 | 0.196 | 0.000 | 0.050 | 0.000 | 0.052 |
| **BAG** | 0.003 | 0.215 | 0.000 | 0.034 |  |  | 0.000 | 0.013 | 0.000 | 0.011 | 0.000 | 0.573 | 0.000 | 0.632 | 0.002 | 0.478 | 0.000 | 0.153 | 0.000 | 0.026 | 0.000 | 0.028 | 0.000 | 0.059 | 0.000 | 0.009 | 0.000 | 0.456 | 0.066 | 2.886 | 0.001 | 0.812 | 0.000 | 0.029 | 0.000 | 0.210 | 0.000 | 0.192 | 0.000 | 0.051 | 0.000 | 0.044 |
| **BAR** | 0.000 | 0.198 | 0.000 | 0.035 | 0.000 | 0.055 |  |  | 0.000 | 0.011 | 0.000 | 0.572 | 0.000 | 0.594 | 0.000 | 0.464 | 0.000 | 0.155 | 0.000 | 0.026 | 0.000 | 0.026 | 0.001 | 0.059 | 0.000 | 0.009 | 0.008 | 0.485 | 0.002 | 1.948 | 0.027 | 0.875 | 0.000 | 0.027 | 0.000 | 0.187 | 0.000 | 0.192 | 0.000 | 0.051 | 0.000 | 0.044 |
| **BOD** | 0.005 | 0.217 | 0.000 | 0.037 | 0.000 | 0.055 | 0.000 | 0.013 |  |  | 0.000 | 0.568 | 0.016 | 0.734 | 0.002 | 0.477 | 0.000 | 0.159 | 0.000 | 0.025 | 0.000 | 0.028 | 0.000 | 0.054 | 0.000 | 0.009 | 0.003 | 0.473 | 0.001 | 2.073 | 0.000 | 0.783 | 0.000 | 0.029 | 0.000 | 0.211 | 0.000 | 0.196 | 0.000 | 0.051 | 0.000 | 0.049 |
| **CAR** | 0.000 | 0.193 | 0.000 | 0.034 | 0.000 | 0.059 | 0.000 | 0.013 | 0.000 | 0.010 |  |  | 0.000 | 0.616 | 0.022 | 0.514 | 0.000 | 0.157 | 0.000 | 0.025 | 0.000 | 0.026 | 0.001 | 0.059 | 0.000 | 0.009 | 0.000 | 0.448 | 0.050 | 2.737 | 0.001 | 0.819 | 0.000 | 0.027 | 0.000 | 0.195 | 0.000 | 0.195 | 0.000 | 0.050 | 0.000 | 0.045 |
| **CHG** | 0.003 | 0.216 | 0.001 | 0.037 | 0.000 | 0.059 | 0.000 | 0.013 | 0.000 | 0.011 | 0.000 | 0.563 |  |  | 0.000 | 0.468 | 0.000 | 0.157 | 0.000 | 0.024 | 0.000 | 0.026 | 0.000 | 0.057 | 0.000 | 0.009 | 0.001 | 0.460 | 0.004 | 2.590 | 0.055 | 0.877 | 0.000 | 0.029 | 0.000 | 0.191 | 0.000 | 0.200 | 0.000 | 0.054 | 0.000 | 0.048 |
| **FAT** | 0.005 | 0.217 | 0.000 | 0.037 | 0.000 | 0.055 | 0.000 | 0.014 | 0.000 | 0.010 | 0.005 | 0.602 | 0.000 | 0.639 |  |  | 0.002 | 0.166 | 0.000 | 0.026 | 0.000 | 0.027 | 0.000 | 0.053 | 0.000 | 0.009 | 0.000 | 0.460 | 0.000 | 1.895 | 0.001 | 0.833 | 0.000 | 0.027 | 0.000 | 0.190 | 0.000 | 0.199 | 0.000 | 0.051 | 0.000 | 0.046 |
| **GSV** | 0.006 | 0.217 | 0.000 | 0.034 | 0.000 | 0.055 | 0.000 | 0.013 | 0.000 | 0.011 | 0.000 | 0.564 | 0.000 | 0.684 | 0.000 | 0.450 |  |  | 0.000 | 0.025 | 0.000 | 0.027 | 0.000 | 0.055 | 0.000 | 0.009 | 0.007 | 0.487 | 0.018 | 2.610 | 0.001 | 0.774 | 0.000 | 0.027 | 0.000 | 0.189 | 0.000 | 0.201 | 0.001 | 0.054 | 0.000 | 0.042 |
| **NIQ** | 0.005 | 0.217 | 0.000 | 0.035 | 0.000 | 0.055 | 0.000 | 0.013 | 0.000 | 0.011 | 0.000 | 0.558 | 0.000 | 0.621 | 0.021 | 0.510 | 0.001 | 0.165 |  |  | 0.000 | 0.028 | 0.000 | 0.058 | 0.000 | 0.009 | 0.000 | 0.444 | 0.000 | 1.986 | 0.005 | 0.853 | 0.000 | 0.028 | 0.000 | 0.197 | 0.000 | 0.209 | 0.000 | 0.051 | 0.000 | 0.049 |
| **PAN** | 0.000 | 0.206 | 0.000 | 0.034 | 0.000 | 0.055 | 0.000 | 0.013 | 0.000 | 0.011 | 0.001 | 0.580 | 0.000 | 0.687 | 0.000 | 0.447 | 0.001 | 0.166 | 0.000 | 0.025 |  |  | 0.000 | 0.055 | 0.000 | 0.009 | 0.010 | 0.486 | 0.001 | 2.013 | 0.009 | 0.851 | 0.000 | 0.029 | 0.000 | 0.187 | 0.007 | 0.223 | 0.001 | 0.054 | 0.000 | 0.045 |
| **PNE** | 0.005 | 0.217 | 0.000 | 0.035 | 0.000 | 0.055 | 0.000 | 0.013 | 0.000 | 0.011 | 0.006 | 0.606 | 0.000 | 0.627 | 0.000 | 0.438 | 0.001 | 0.166 | 0.000 | 0.026 | 0.000 | 0.028 |  |  | 0.000 | 0.009 | 0.000 | 0.454 | 0.075 | 2.939 | 0.000 | 0.791 | 0.000 | 0.027 | 0.000 | 0.211 | 0.000 | 0.193 | 0.000 | 0.051 | 0.000 | 0.043 |
| **POT** | 0.000 | 0.198 | 0.000 | 0.037 | 0.000 | 0.054 | 0.000 | 0.013 | 0.000 | 0.010 | 0.000 | 0.557 | 0.011 | 0.732 | 0.001 | 0.471 | 0.000 | 0.164 | 0.000 | 0.025 | 0.000 | 0.028 | 0.000 | 0.058 |  |  | 0.000 | 0.451 | 0.007 | 2.496 | 0.000 | 0.793 | 0.000 | 0.027 | 0.000 | 0.189 | 0.008 | 0.232 | 0.000 | 0.050 | 0.000 | 0.046 |
| **PTU** | 0.005 | 0.217 | 0.000 | 0.034 | 0.000 | 0.055 | 0.000 | 0.013 | 0.000 | 0.010 | 0.001 | 0.575 | 0.000 | 0.665 | 0.000 | 0.444 | 0.000 | 0.157 | 0.000 | 0.025 | 0.000 | 0.026 | 0.000 | 0.053 | 0.000 | 0.009 |  |  | 0.067 | 2.881 | 0.041 | 0.882 | 0.000 | 0.027 | 0.000 | 0.185 | 0.000 | 0.199 | 0.000 | 0.050 | 0.000 | 0.046 |
| **SAF** | 0.008 | 0.217 | 0.000 | 0.033 | 0.000 | 0.055 | 0.000 | 0.012 | 0.000 | 0.011 | 0.016 | 0.606 | 0.028 | 0.734 | 0.000 | 0.454 | 0.000 | 0.154 | 0.000 | 0.025 | 0.000 | 0.028 | 0.000 | 0.058 | 0.000 | 0.009 | 0.001 | 0.456 |  |  | 0.000 | 0.775 | 0.000 | 0.027 | 0.000 | 0.192 | 0.000 | 0.197 | 0.000 | 0.050 | 0.000 | 0.046 |
| **SCA** | 0.000 | 0.193 | 0.000 | 0.035 | 0.000 | 0.055 | 0.000 | 0.014 | 0.000 | 0.010 | 0.000 | 0.564 | 0.013 | 0.734 | 0.001 | 0.466 | 0.000 | 0.151 | 0.000 | 0.025 | 0.000 | 0.028 | 0.000 | 0.054 | 0.000 | 0.009 | 0.018 | 0.488 | 0.003 | 1.805 |  |  | 0.000 | 0.029 | 0.000 | 0.204 | 0.000 | 0.196 | 0.000 | 0.052 | 0.000 | 0.051 |
| **SDO** | 0.000 | 0.196 | 0.000 | 0.035 | 0.000 | 0.054 | 0.000 | 0.013 | 0.000 | 0.011 | 0.000 | 0.542 | 0.000 | 0.628 | 0.000 | 0.459 | 0.000 | 0.156 | 0.000 | 0.025 | 0.000 | 0.028 | 0.000 | 0.053 | 0.000 | 0.009 | 0.015 | 0.488 | 0.001 | 1.870 | 0.020 | 0.877 |  |  | 0.000 | 0.211 | 0.000 | 0.208 | 0.000 | 0.051 | 0.000 | 0.050 |
| **SEC** | 0.000 | 0.202 | 0.000 | 0.036 | 0.000 | 0.059 | 0.000 | 0.014 | 0.000 | 0.011 | 0.000 | 0.542 | 0.014 | 0.733 | 0.000 | 0.414 | 0.000 | 0.155 | 0.000 | 0.026 | 0.000 | 0.026 | 0.000 | 0.054 | 0.000 | 0.009 | 0.000 | 0.452 | 0.001 | 1.758 | 0.002 | 0.831 | 0.000 | 0.028 |  |  | 0.007 | 0.226 | 0.000 | 0.050 | 0.000 | 0.045 |
| **STZ** | 0.006 | 0.217 | 0.000 | 0.035 | 0.000 | 0.059 | 0.000 | 0.012 | 0.000 | 0.011 | 0.009 | 0.596 | 0.000 | 0.618 | 0.021 | 0.514 | 0.000 | 0.158 | 0.000 | 0.025 | 0.000 | 0.026 | 0.000 | 0.054 | 0.000 | 0.009 | 0.020 | 0.487 | 0.000 | 2.175 | 0.000 | 0.802 | 0.000 | 0.029 | 0.000 | 0.188 |  |  | 0.000 | 0.050 | 0.000 | 0.047 |
| **SUM** | 0.000 | 0.202 | 0.000 | 0.034 | 0.000 | 0.055 | 0.000 | 0.013 | 0.000 | 0.011 | 0.000 | 0.550 | 0.000 | 0.650 | 0.025 | 0.513 | 0.000 | 0.155 | 0.000 | 0.025 | 0.000 | 0.028 | 0.000 | 0.053 | 0.000 | 0.009 | 0.000 | 0.433 | 0.000 | 2.555 | 0.003 | 0.823 | 0.000 | 0.027 | 0.000 | 0.187 | 0.000 | 0.189 |  |  | 0.000 | 0.048 |
| **VIB** | 0.000 | 0.198 | 0.000 | 0.034 | 0.000 | 0.057 | 0.000 | 0.013 | 0.000 | 0.010 | 0.009 | 0.605 | 0.000 | 0.666 | 0.001 | 0.478 | 0.000 | 0.155 | 0.000 | 0.026 | 0.000 | 0.026 | 0.000 | 0.057 | 0.000 | 0.009 | 0.000 | 0.440 | 0.000 | 2.448 | 0.000 | 0.828 | 0.000 | 0.027 | 0.000 | 0.194 | 0.017 | 0.234 | 0.000 | 0.054 |  |  |

**Table S9** Uncertainty of the modeling components from ecological niche modeling predictions for *Tabebuia aurea* as revealed by hierarchical ANOVA. SS: sum of square.

| **Source of variation** | **Median SS** | **Minimun** | **Maximum** |
| --- | --- | --- | --- |
| **TIME** | 0.25 | 0.00 | 0.94 |
| **AOGCM** | 0.35 | 0.02 | 0.96 |
| **ENM** | 0.18 | 0.004 | 0.81 |
| **AOGCM x ENM** | 0.09 | 0.003 | 0.40 |

**Table S10** Classification of the 52 predictive maps following the three general scenarios of the distribution dynamics through the time: range stability (S); retraction (R) and expansion (E).

|  |  | **Range Size** | | **Range Shift** | |
| --- | --- | --- | --- | --- | --- |
| **AOGCM** | **ENM** | **21 ka** | **0 ka** | **shift** | ***H*** |
| CCSM | BioClim | 1357 | 1629 | 272 | E |
| CCSM | ENFA | 2321 | 2609 | 288 | E |
| CCSM | EuclidDist | 1419 | 1840 | 421 | E |
| CCSM | FDA | 1376 | 1550 | 174 | E |
| CCSM | GAM | 1575 | 1578 | 3 | S |
| CCSM | GBM | 1249 | 1477 | 228 | E |
| CCSM | GLM | 1597 | 1688 | 91 | S |
| CCSM | GowerDist | 1485 | 1931 | 446 | E |
| CCSM | MahalDist | 1978 | 2151 | 173 | E |
| CCSM | MARS | 1349 | 1477 | 128 | E |
| CCSM | MaxEnt | 1471 | 1527 | 56 | S |
| CCSM | NNet | 1411 | 1700 | 289 | E |
| CCSM | RndFor | 1278 | 1536 | 258 | E |
| CNRM | BioClim | 1117 | 1601 | 484 | E |
| CNRM | ENFA | 2601 | 2599 | -2 | S |
| CNRM | EuclidDist | 1912 | 1809 | -103 | S |
| CNRM | FDA | 1620 | 1507 | -113 | R |
| CNRM | GAM | 1700 | 1594 | -106 | S |
| CNRM | GBM | 1325 | 1435 | 110 | S |
| CNRM | GLM | 1464 | 1573 | 109 | S |
| CNRM | GowerDist | 1912 | 1764 | -148 | R |
| CNRM | MahalDist | 1780 | 1719 | -61 | S |
| CNRM | MARS | 1569 | 1489 | -80 | S |
| CNRM | MaxEnt | 1387 | 1482 | 95 | S |
| CNRM | NNet | 1213 | 1477 | 264 | E |
| CNRM | RndFor | 1504 | 1589 | 85 | S |
| MIROC | BioClim | 1642 | 1855 | 213 | E |
| MIROC | ENFA | 2527 | 2599 | 72 | S |
| MIROC | EuclidDist | 1937 | 2098 | 161 | E |
| MIROC | FDA | 1563 | 1934 | 371 | E |
| MIROC | GAM | 1716 | 1749 | 33 | S |
| MIROC | GBM | 1310 | 1652 | 342 | E |
| MIROC | GLM | 1867 | 1764 | -103 | S |
| MIROC | GowerDist | 2151 | 2421 | 270 | E |
| MIROC | MahalDist | 2156 | 2492 | 336 | E |
| MIROC | MARS | 1372 | 1733 | 361 | E |
| MIROC | MaxEnt | 1290 | 1614 | 324 | E |
| MIROC | NNet | 1522 | 1561 | 39 | S |
| MIROC | RndFor | 1448 | 1972 | 524 | E |
| MRI | BioClim | 1007 | 2135 | 1128 | E |
| MRI | ENFA | 693 | 2819 | 2126 | E |
| MRI | EuclidDist | 1595 | 2112 | 517 | E |
| MRI | FDA | 211 | 1744 | 1533 | E |
| MRI | GAM | 51 | 1739 | 1688 | E |
| MRI | GBM | 116 | 1585 | 1469 | E |
| MRI | GLM | 41 | 1690 | 1649 | E |
| MRI | GowerDist | 784 | 1940 | 1156 | E |
| MRI | MahalDist | 1201 | 2153 | 952 | E |
| MRI | MARS | 58 | 1642 | 1584 | E |
| MRI | MaxEnt | 52 | 1513 | 1461 | E |
| MRI | NNet | 155 | 1657 | 1502 | E |
| MRI | RndFor | 591 | 1695 | 1104 | E |

Legend:

AOGCM: coupled atmosphere-ocean general circulation models (for details see Table S3);

ENM: algorithms (for details see Table S4);

Range size: geographical range size predicted by each combination of algorithm and AOGCM in two time periods (LGM – 21 ka and present-day – 0 ka). Unit is number of grid cells (0.5^o^ resolution) climatically suitable for *T. aurea*;

Range shift: "shift" – difference of range size between time period (21-0 ka);

*"H"* – hypothesis from map classification matching the scenarios range expansion ("E"; shift > 110); range retraction ("R"; shift < -110). or range stability ("S"; -110 < shift < 110).

**Table S11** Profile of genetic parameters obtained from the 2,000 simulations of six different demographic scenarios using the software BayeSSC, for chloroplast and ITS sequences. *h* – haplotype diversity; π – nucleotide diversity. See Fig 2 for details about the demographical scenarios and simulations. Observed genetic parameter means for chloroplast DNA, *h* = 0.22, π = 0.0093; for ITS, *h* = 0.089, π = 0.0006.

|  | **Chloroplast** | | | | **ITS** | | | |
| --- | --- | --- | --- | --- | --- | --- | --- | --- |
| **Models** | *h* | | π | | *h* | | π | |
|  | ***Range*** | ***Mean (SD)*** | ***Range*** | ***Mean (SD)*** | ***Range*** | ***Mean (SD)*** | ***Range*** | ***Mean (SD)*** |
| **Stability** | 0.325 – 0.929 | 0.785 (0.098) | 0.0134 – 0.0488 | 0.0220 (0.0011) | 0.251 – 0.867 | 0.417 (0.187) | 0.0008 - 0.0038 | 0.0012 (0.0052) |
| **Retraction** | 0.0001 – 0.109 | 0.043 (0.100) | 8.7E-5 – 0.0004 | 7.7 E-4 (0.0001) | 0.005 – 0.079 | 0.017 (0.056) | 2.1E-6 - 0.00004 | 7.9E-6 (4.7E-6) |
| **Expansion** | 0.016 – 0.499 | 0.293 (0.151) | 0.0002 – 0.0130 | 0.0049 (0.0109) | 0.027 – 0.624 | 0.480 (0.420) | 2.0E-5 – 0.0030 | 0.0009 (0.0017) |
| **Multiple Refugia** | 0.287 – 0.773 | 0.436 (0.173) | 0.0101– 0.0413 | 0.0185 (0.0235) | 0.119- 0.893 | 0.379 (0.203) | 0.0004 - 0.0084 | 0.0034 (0.0026) |
