# Supplementary material for: Relaxed random walk model coupled with ecological niche modeling unravel the dispersal dynamics of a Neotropical savanna tree species in the deeper Quaternary
Source: Front Plant Sci. 2015 Aug 25;6:653. doi: 10.3389/fpls.2015.00653 (PMC4548090; doi:10.3389/fpls.2015.00653)
Supplement: Supplementary file 2 [file Table2.DOC]

Frontiers in Plant Science

Supporting Information

**Relaxed random walk model coupled with ecological niche modelling unravel the dispersal dynamics of a Neotropical savanna tree species in the deeper Quaternary**

Rosane G. Collevatti, Levi Carina Terribile, Suelen G. Rabelo, Matheus S. Lima-Ribeiro

**Appendix S2 Figures**

**
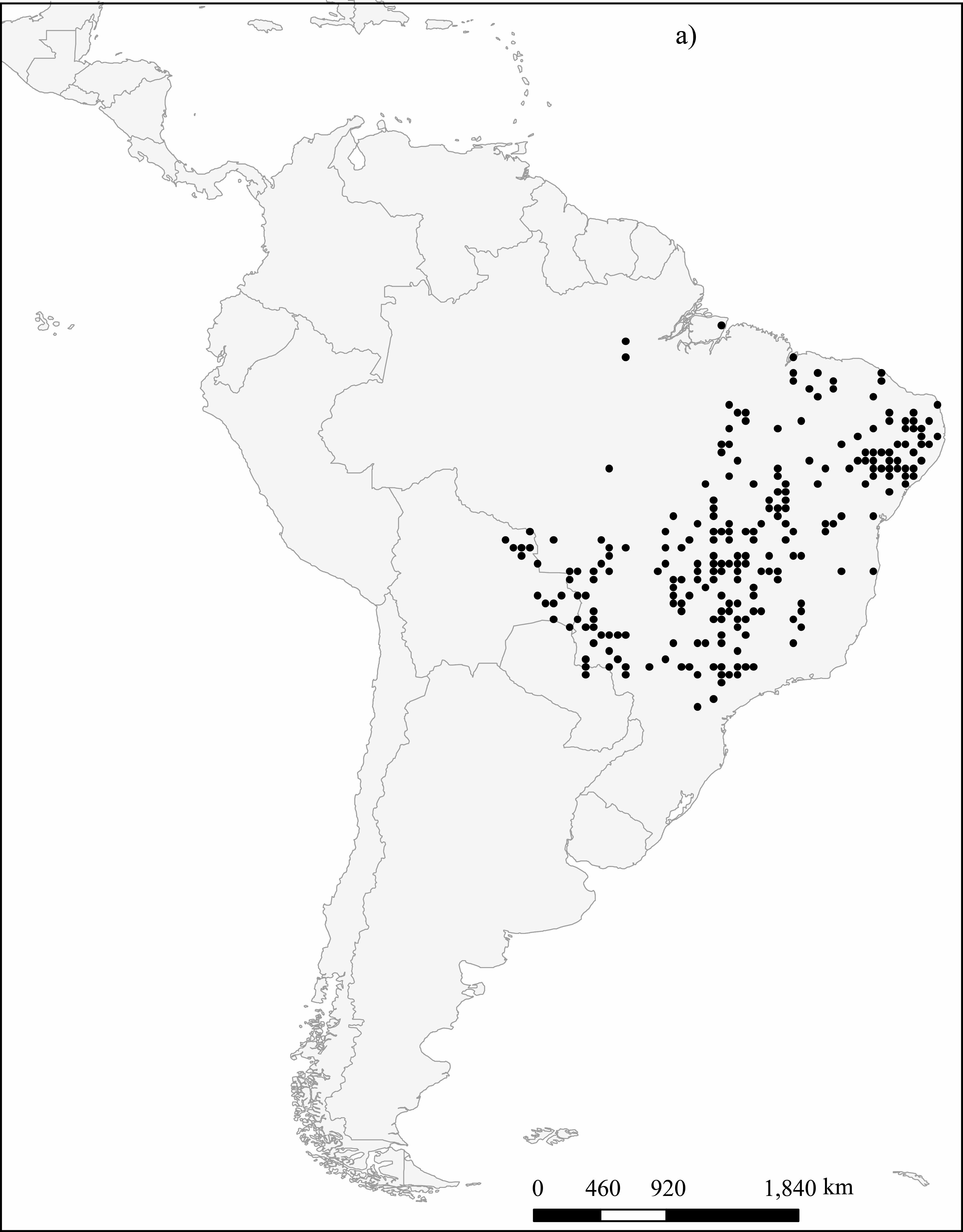
**

**Fig S1** Current geographical distribution of *Tabebuia aurea* across the Neotropics based on 237 occurrence records used in ecological niche modeling.

**
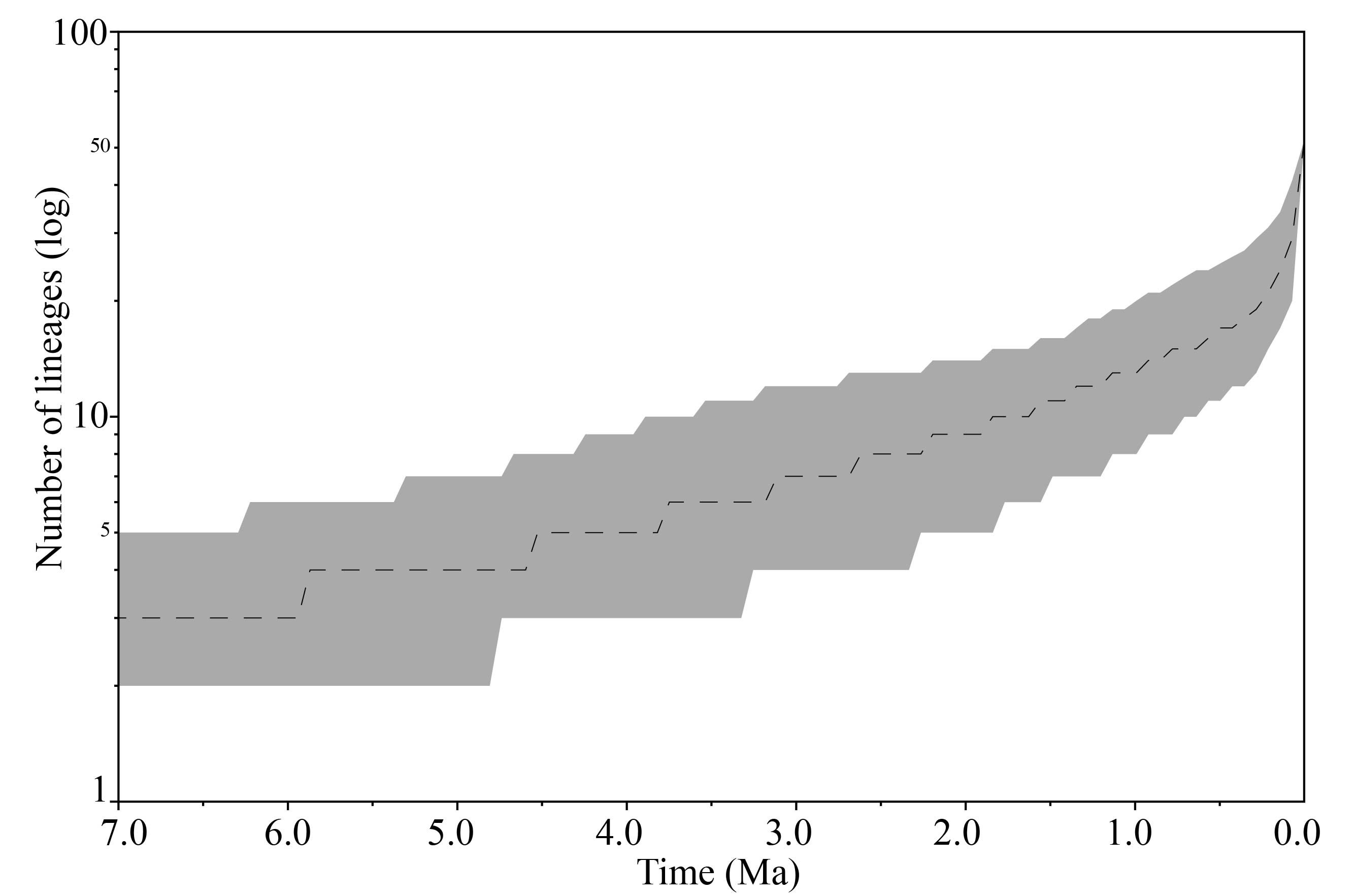
**

**Fig S2** Diversification time of *Tabebuia aurea* lineages using Lineage Through Time (LTT) analysis.


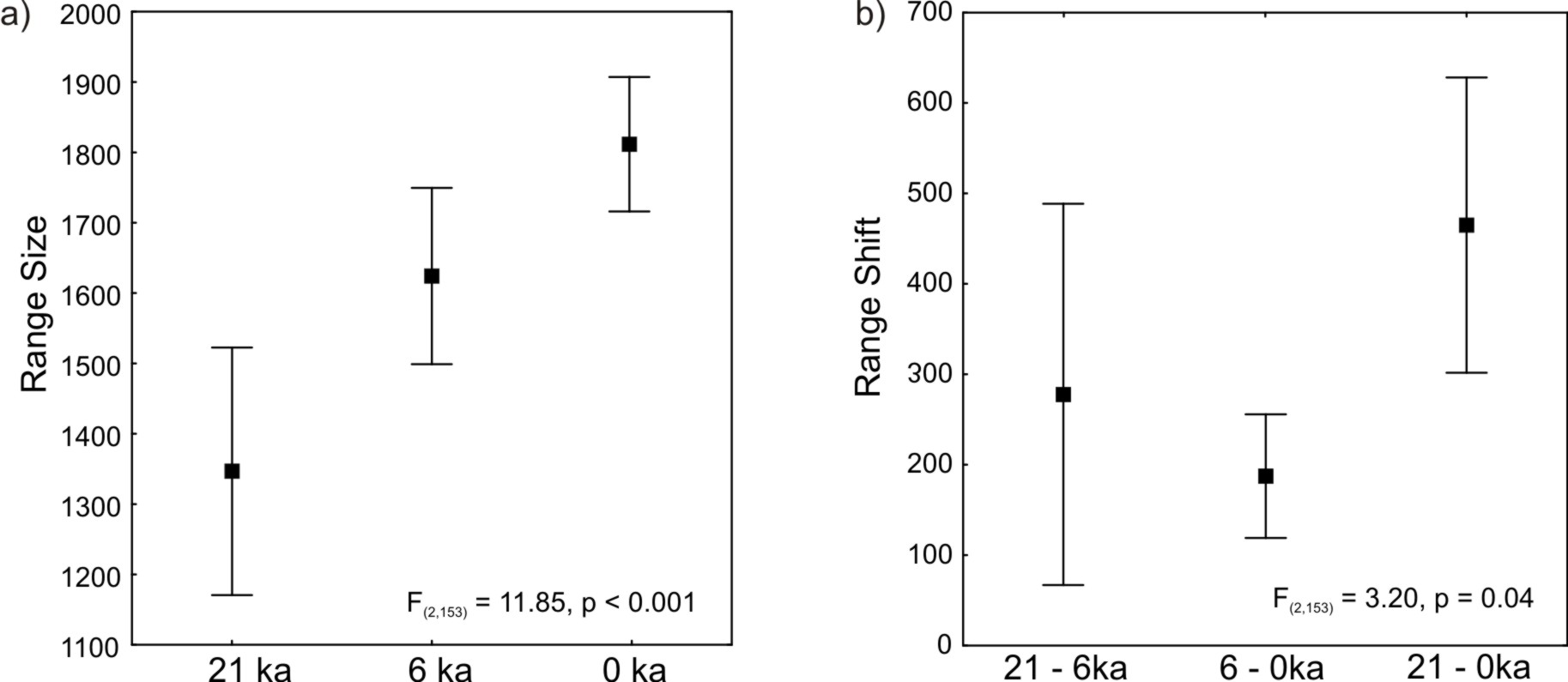


**Fig S3** Average and 0.95 confidence interval among the 52 maps of **(a)** range size and **(b)** shift (difference of range size among time periods in number of cells) predicted for *Tabebuia aurea* at LGM (21 ka), mid-Holocene (6 ka), and present-day (0 ka). F and p values are Anova analysis showing significant difference among time periods). Note that a general scenario of range expansion though time (positive range shifts) is supported.


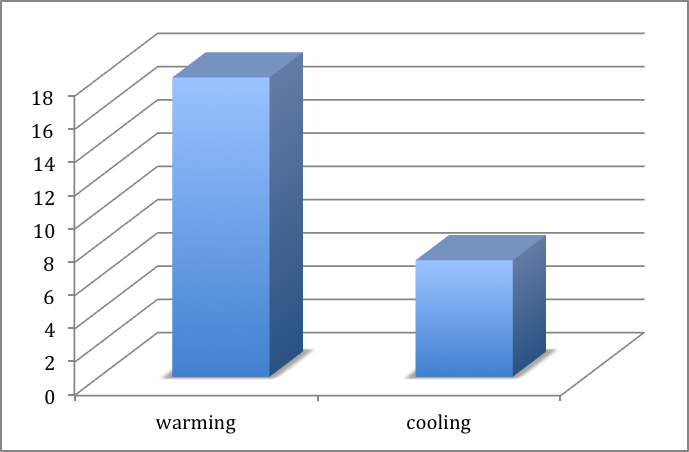

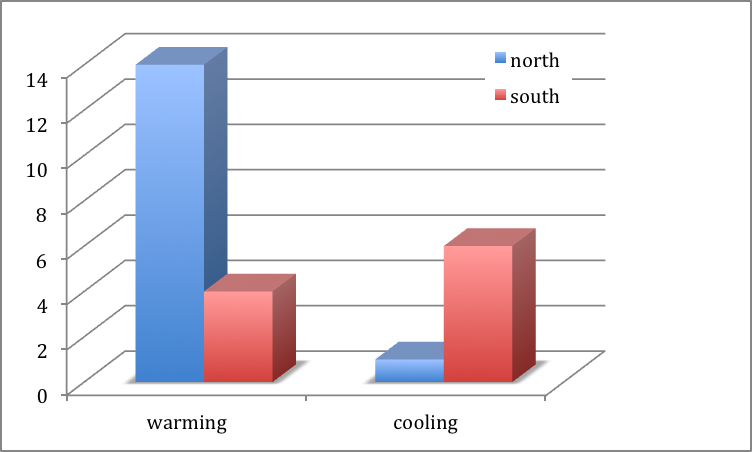


(**a**) (**b**)

**Fig S4** Number of dispersal events from the phylogeographic reconstruction tree (see Fig 5 in main text) matching periods of warming and cooling (**a**) and their directions (toward north or south; **b**).


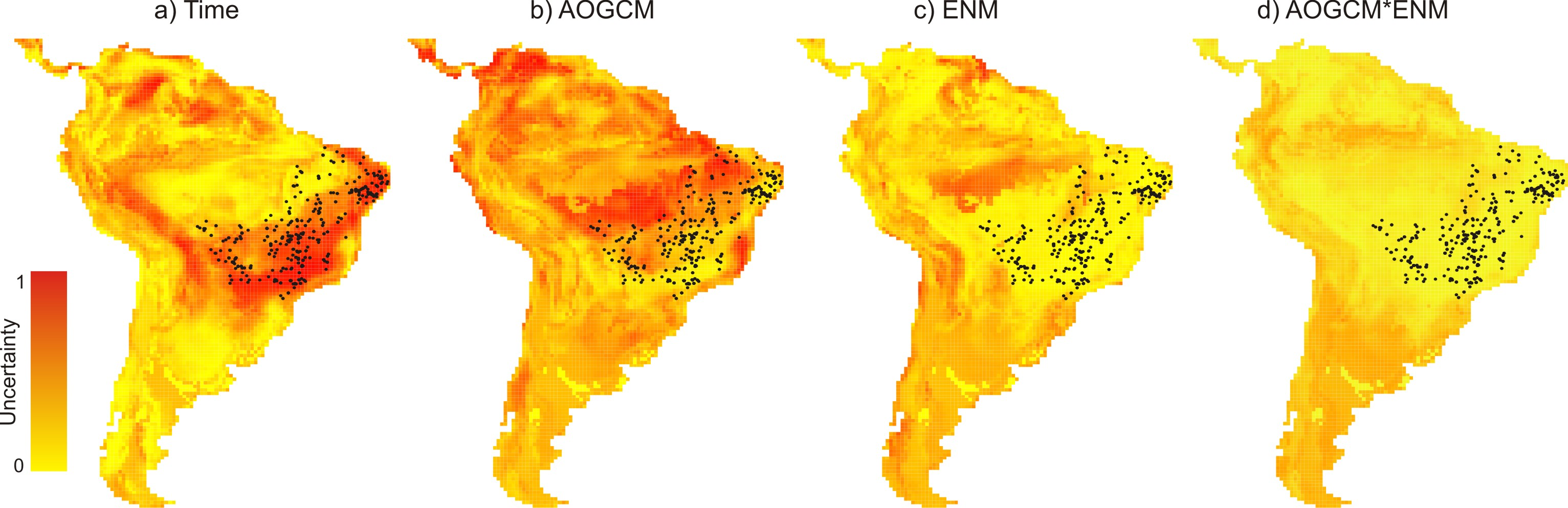


**Fig S5** Maps of uncertainty (relative sum of squares) for the modelling components of *Tabebuia aurea*, **(a)** Time, **(b)** Atmosphere-Ocean Global Circulation Models (AOGCMs), **(c)** Ecological Niche Models (ENMs), (**d**) Interaction AOGCM*ENM. Black dots are occurrence records used in ecological niche modelling.


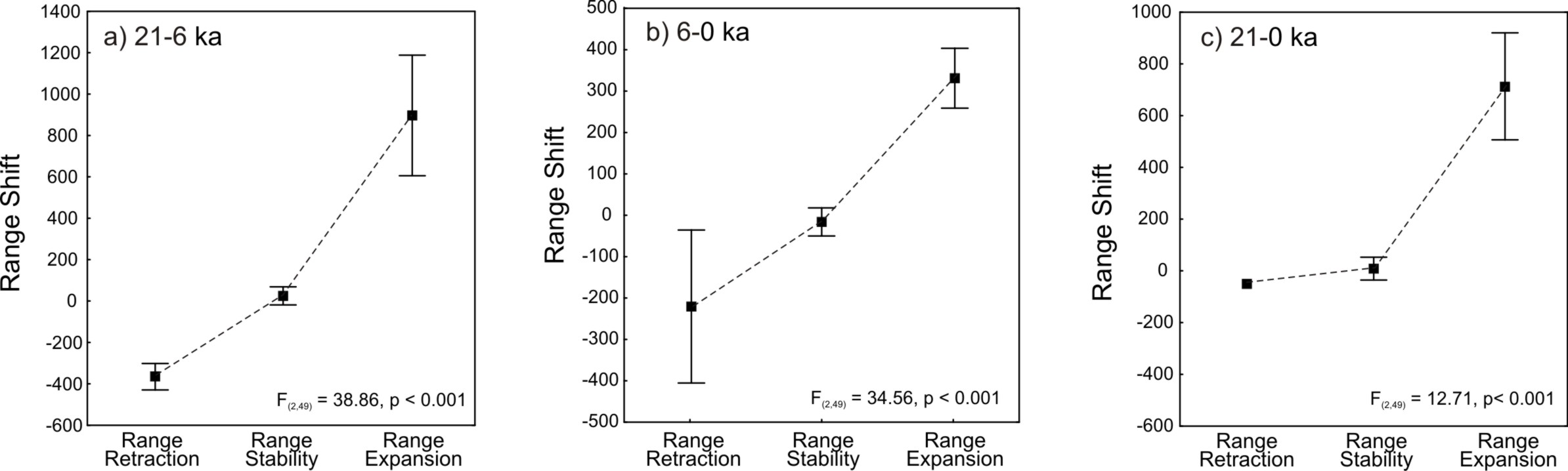


**Fig S6** Average and 0.95 confidence interval of range shift predicted by palaeodistribution modelling for *Tabebuia aurea* in each scenario and time slice: **(a)** 21kyr -6kyr - range size at 6 ka minus 21 ka; **(b)** 6kyr - 0kyr - range size at 0 ka minus 6 ka; and **(c)** 21kyr - 0kyr - range size at 0 ka minus 21 ka. In (c), the range retraction does not present confidence interval because just two maps predicted that scenario between LGM and present. F and p values are Anova analysis showing significant difference among time periods). Note that the average of range shift predicted by 52 maps follows systematically the expected by each general scenario (positive range shift for range expansion, negative range shift for range retraction, and no variation for range stability).
