# Supplementary material for: Relaxed random walk model coupled with ecological niche modeling unravel the dispersal dynamics of a Neotropical savanna tree species in the deeper Quaternary
Source: Front Plant Sci. 2015 Aug 25;6:653. doi: 10.3389/fpls.2015.00653 (PMC4548090; doi:10.3389/fpls.2015.00653)
Supplement: Supplementary file 3 [file Table3.DOCX]

TaureatrnGtrnSGenbank2.sqn AGE03 KM053413

TaureatrnGtrnSGenbank2.sqn AGE06 KM053414

TaureatrnGtrnSGenbank2.sqn AGE08 KM053415

TaureatrnGtrnSGenbank2.sqn AGE13 KM053416

TaureatrnGtrnSGenbank2.sqn AGE17 KM053417

TaureatrnGtrnSGenbank2.sqn AGE18 KM053418

TaureatrnGtrnSGenbank2.sqn AGE19 KM053419

TaureatrnGtrnSGenbank2.sqn AGE20 KM053420

TaureatrnGtrnSGenbank2.sqn AGE23 KM053421

TaureatrnGtrnSGenbank2.sqn AGE28 KM053422

TaureatrnGtrnSGenbank2.sqn AGE30 KM053423

TaureatrnGtrnSGenbank2.sqn AGE31 KM053424

TaureatrnGtrnSGenbank2.sqn AGE32 KM053425

TaureatrnGtrnSGenbank2.sqn AGE33 KM053426

TaureatrnGtrnSGenbank2.sqn AGE35 KM053427

TaureatrnGtrnSGenbank2.sqn AGE36 KM053428

TaureatrnGtrnSGenbank2.sqn ARA01 KM053429

TaureatrnGtrnSGenbank2.sqn ARA02 KM053430

TaureatrnGtrnSGenbank2.sqn ARA04 KM053431

TaureatrnGtrnSGenbank2.sqn ARA05 KM053432

TaureatrnGtrnSGenbank2.sqn ARA06 KM053433

TaureatrnGtrnSGenbank2.sqn ARA07 KM053434

TaureatrnGtrnSGenbank2.sqn ARA08 KM053435

TaureatrnGtrnSGenbank2.sqn ARA09 KM053436

TaureatrnGtrnSGenbank2.sqn ARA10 KM053437

TaureatrnGtrnSGenbank2.sqn ARA11 KM053438

TaureatrnGtrnSGenbank2.sqn ARA12 KM053439

TaureatrnGtrnSGenbank2.sqn ARA13 KM053440

TaureatrnGtrnSGenbank2.sqn ARA14 KM053441

TaureatrnGtrnSGenbank2.sqn BAG01 KM053442

TaureatrnGtrnSGenbank2.sqn BAG02 KM053443

TaureatrnGtrnSGenbank2.sqn BAG04 KM053444

TaureatrnGtrnSGenbank2.sqn BAG05 KM053445

TaureatrnGtrnSGenbank2.sqn BAG07 KM053446

TaureatrnGtrnSGenbank2.sqn BAG08 KM053447

TaureatrnGtrnSGenbank2.sqn BAG09 KM053448

TaureatrnGtrnSGenbank2.sqn BAG10 KM053449

TaureatrnGtrnSGenbank2.sqn BAG11 KM053450

TaureatrnGtrnSGenbank2.sqn BAG13 KM053451

TaureatrnGtrnSGenbank2.sqn BAG15 KM053452

TaureatrnGtrnSGenbank2.sqn BAG16 KM053453

TaureatrnGtrnSGenbank2.sqn BAG17 KM053454

TaureatrnGtrnSGenbank2.sqn BAG18 KM053455

TaureatrnGtrnSGenbank2.sqn BAG19 KM053456

TaureatrnGtrnSGenbank2.sqn BAG20 KM053457

TaureatrnGtrnSGenbank2.sqn BAG21 KM053458

TaureatrnGtrnSGenbank2.sqn BAG27 KM053459

TaureatrnGtrnSGenbank2.sqn BAR01 KM053460

TaureatrnGtrnSGenbank2.sqn BAR02 KM053461

TaureatrnGtrnSGenbank2.sqn BAR03 KM053462

TaureatrnGtrnSGenbank2.sqn BAR04 KM053463

TaureatrnGtrnSGenbank2.sqn BAR05 KM053464

TaureatrnGtrnSGenbank2.sqn BAR09 KM053465

TaureatrnGtrnSGenbank2.sqn BAR10 KM053466

TaureatrnGtrnSGenbank2.sqn BAR11 KM053467

TaureatrnGtrnSGenbank2.sqn BAR12 KM053468

TaureatrnGtrnSGenbank2.sqn BAR13 KM053469

TaureatrnGtrnSGenbank2.sqn BAR15 KM053470

TaureatrnGtrnSGenbank2.sqn BAR16 KM053471

TaureatrnGtrnSGenbank2.sqn BOD01 KM053472

TaureatrnGtrnSGenbank2.sqn BOD02 KM053473

TaureatrnGtrnSGenbank2.sqn BOD03 KM053474

TaureatrnGtrnSGenbank2.sqn BOD06 KM053475

TaureatrnGtrnSGenbank2.sqn BOD07 KM053476

TaureatrnGtrnSGenbank2.sqn BOD08 KM053477

TaureatrnGtrnSGenbank2.sqn BOD09 KM053478

TaureatrnGtrnSGenbank2.sqn BOD10 KM053479

TaureatrnGtrnSGenbank2.sqn BOD11 KM053480

TaureatrnGtrnSGenbank2.sqn BOD12 KM053481

TaureatrnGtrnSGenbank2.sqn BOD14 KM053482

TaureatrnGtrnSGenbank2.sqn BOD15 KM053483

TaureatrnGtrnSGenbank2.sqn BOD16 KM053484

TaureatrnGtrnSGenbank2.sqn BOD17 KM053485

TaureatrnGtrnSGenbank2.sqn BOD18 KM053486

TaureatrnGtrnSGenbank2.sqn BOD19 KM053487

TaureatrnGtrnSGenbank2.sqn CAR01 KM053488

TaureatrnGtrnSGenbank2.sqn CAR02 KM053489

TaureatrnGtrnSGenbank2.sqn CAR03 KM053490

TaureatrnGtrnSGenbank2.sqn CAR04 KM053491

TaureatrnGtrnSGenbank2.sqn CAR05 KM053492

TaureatrnGtrnSGenbank2.sqn CAR06 KM053493

TaureatrnGtrnSGenbank2.sqn CAR07 KM053494

TaureatrnGtrnSGenbank2.sqn CAR08 KM053495

TaureatrnGtrnSGenbank2.sqn CAR09 KM053496

TaureatrnGtrnSGenbank2.sqn CAR10 KM053497

TaureatrnGtrnSGenbank2.sqn CAR11 KM053498

TaureatrnGtrnSGenbank2.sqn CAR12 KM053499

TaureatrnGtrnSGenbank2.sqn CAR13 KM053500

TaureatrnGtrnSGenbank2.sqn CAR14 KM053501

TaureatrnGtrnSGenbank2.sqn CAR15 KM053502

TaureatrnGtrnSGenbank2.sqn CAR16 KM053503

TaureatrnGtrnSGenbank2.sqn CHG01 KM053504

TaureatrnGtrnSGenbank2.sqn CHG02 KM053505

TaureatrnGtrnSGenbank2.sqn CHG03 KM053506

TaureatrnGtrnSGenbank2.sqn CHG04 KM053507

TaureatrnGtrnSGenbank2.sqn CHG05 KM053508

TaureatrnGtrnSGenbank2.sqn CHG06 KM053509

TaureatrnGtrnSGenbank2.sqn CHG07 KM053510

TaureatrnGtrnSGenbank2.sqn CHG09 KM053511

TaureatrnGtrnSGenbank2.sqn CHG10 KM053512

TaureatrnGtrnSGenbank2.sqn CHG11 KM053513

TaureatrnGtrnSGenbank2.sqn CHG12 KM053514

TaureatrnGtrnSGenbank2.sqn CHG13 KM053515

TaureatrnGtrnSGenbank2.sqn CHG14 KM053516

TaureatrnGtrnSGenbank2.sqn CHG15 KM053517

TaureatrnGtrnSGenbank2.sqn CHG16 KM053518

TaureatrnGtrnSGenbank2.sqn FAT01 KM053519

TaureatrnGtrnSGenbank2.sqn FAT02 KM053520

TaureatrnGtrnSGenbank2.sqn FAT03 KM053521

TaureatrnGtrnSGenbank2.sqn FAT04 KM053522

TaureatrnGtrnSGenbank2.sqn FAT05 KM053523

TaureatrnGtrnSGenbank2.sqn FAT06 KM053524

TaureatrnGtrnSGenbank2.sqn FAT11 KM053525

TaureatrnGtrnSGenbank2.sqn FAT13 KM053526

TaureatrnGtrnSGenbank2.sqn FAT14 KM053527

TaureatrnGtrnSGenbank2.sqn FAT15 KM053528

TaureatrnGtrnSGenbank2.sqn FAT16 KM053529

TaureatrnGtrnSGenbank2.sqn FAT20 KM053530

TaureatrnGtrnSGenbank2.sqn FAT21 KM053531

TaureatrnGtrnSGenbank2.sqn FAT22 KM053532

TaureatrnGtrnSGenbank2.sqn FAT23 KM053533

TaureatrnGtrnSGenbank2.sqn FAT31 KM053534

TaureatrnGtrnSGenbank2.sqn FAT33 KM053535

TaureatrnGtrnSGenbank2.sqn FAT35 KM053536

TaureatrnGtrnSGenbank2.sqn FAT36 KM053537

TaureatrnGtrnSGenbank2.sqn FAT37 KM053538

TaureatrnGtrnSGenbank2.sqn FAT38 KM053539

TaureatrnGtrnSGenbank2.sqn GSV01 KM053540

TaureatrnGtrnSGenbank2.sqn GSV02 KM053541

TaureatrnGtrnSGenbank2.sqn GSV03 KM053542

TaureatrnGtrnSGenbank2.sqn GSV04 KM053543

TaureatrnGtrnSGenbank2.sqn GSV05 KM053544

TaureatrnGtrnSGenbank2.sqn GSV06 KM053545

TaureatrnGtrnSGenbank2.sqn GSV07 KM053546

TaureatrnGtrnSGenbank2.sqn GSV08 KM053547

TaureatrnGtrnSGenbank2.sqn NIQ01 KM053548

TaureatrnGtrnSGenbank2.sqn NIQ02 KM053549

TaureatrnGtrnSGenbank2.sqn NIQ03 KM053550

TaureatrnGtrnSGenbank2.sqn NIQ04 KM053551

TaureatrnGtrnSGenbank2.sqn NIQ05 KM053552

TaureatrnGtrnSGenbank2.sqn NIQ08 KM053553

TaureatrnGtrnSGenbank2.sqn NIQ06 KM053554

TaureatrnGtrnSGenbank2.sqn NIQ10 KM053555

TaureatrnGtrnSGenbank2.sqn NIQ14 KM053556

TaureatrnGtrnSGenbank2.sqn NIQ17 KM053557

TaureatrnGtrnSGenbank2.sqn NIQ19 KM053558

TaureatrnGtrnSGenbank2.sqn NIQ22 KM053559

TaureatrnGtrnSGenbank2.sqn NIQ25 KM053560

TaureatrnGtrnSGenbank2.sqn NIQ26 KM053561

TaureatrnGtrnSGenbank2.sqn PAN01 KM053562

TaureatrnGtrnSGenbank2.sqn PAN02 KM053563

TaureatrnGtrnSGenbank2.sqn PAN03 KM053564

TaureatrnGtrnSGenbank2.sqn PAN04 KM053565

TaureatrnGtrnSGenbank2.sqn PAN05 KM053566

TaureatrnGtrnSGenbank2.sqn PAN06 KM053567

TaureatrnGtrnSGenbank2.sqn PAN07 KM053568

TaureatrnGtrnSGenbank2.sqn PAN08 KM053569

TaureatrnGtrnSGenbank2.sqn PAN09 KM053570

TaureatrnGtrnSGenbank2.sqn PAN10 KM053571

TaureatrnGtrnSGenbank2.sqn PAN11 KM053572

TaureatrnGtrnSGenbank2.sqn PAN12 KM053573

TaureatrnGtrnSGenbank2.sqn PAN13 KM053574

TaureatrnGtrnSGenbank2.sqn PAN14 KM053575

TaureatrnGtrnSGenbank2.sqn PAN15 KM053576

TaureatrnGtrnSGenbank2.sqn PAN16 KM053577

TaureatrnGtrnSGenbank2.sqn PNE01 KM053578

TaureatrnGtrnSGenbank2.sqn PNE02 KM053579

TaureatrnGtrnSGenbank2.sqn PNE03 KM053580

TaureatrnGtrnSGenbank2.sqn PNE04 KM053581

TaureatrnGtrnSGenbank2.sqn PNE05 KM053582

TaureatrnGtrnSGenbank2.sqn PNE06 KM053583

TaureatrnGtrnSGenbank2.sqn PNE07 KM053584

TaureatrnGtrnSGenbank2.sqn PNE08 KM053585

TaureatrnGtrnSGenbank2.sqn PNE09 KM053586

TaureatrnGtrnSGenbank2.sqn PNE10 KM053587

TaureatrnGtrnSGenbank2.sqn PNE11 KM053588

TaureatrnGtrnSGenbank2.sqn PNE12 KM053589

TaureatrnGtrnSGenbank2.sqn PNE13 KM053590

TaureatrnGtrnSGenbank2.sqn PNE14 KM053591

TaureatrnGtrnSGenbank2.sqn PNE15 KM053592

TaureatrnGtrnSGenbank2.sqn PNE16 KM053593

TaureatrnGtrnSGenbank2.sqn POT01 KM053594

TaureatrnGtrnSGenbank2.sqn POT02 KM053595

TaureatrnGtrnSGenbank2.sqn POT03 KM053596

TaureatrnGtrnSGenbank2.sqn POT04 KM053597

TaureatrnGtrnSGenbank2.sqn POT05 KM053598

TaureatrnGtrnSGenbank2.sqn POT06 KM053599

TaureatrnGtrnSGenbank2.sqn POT07 KM053600

TaureatrnGtrnSGenbank2.sqn POT08 KM053601

TaureatrnGtrnSGenbank2.sqn POT09 KM053602

TaureatrnGtrnSGenbank2.sqn POT10 KM053603

TaureatrnGtrnSGenbank2.sqn POT11 KM053604

TaureatrnGtrnSGenbank2.sqn POT12 KM053605

TaureatrnGtrnSGenbank2.sqn POT13 KM053606

TaureatrnGtrnSGenbank2.sqn POT14 KM053607

TaureatrnGtrnSGenbank2.sqn POT15 KM053608

TaureatrnGtrnSGenbank2.sqn POT16 KM053609

TaureatrnGtrnSGenbank2.sqn PTU01 KM053610

TaureatrnGtrnSGenbank2.sqn PTU02 KM053611

TaureatrnGtrnSGenbank2.sqn PTU03 KM053612

TaureatrnGtrnSGenbank2.sqn PTU04 KM053613

TaureatrnGtrnSGenbank2.sqn PTU05 KM053614

TaureatrnGtrnSGenbank2.sqn PTU06 KM053615

TaureatrnGtrnSGenbank2.sqn PTU07 KM053616

TaureatrnGtrnSGenbank2.sqn PTU09 KM053617

TaureatrnGtrnSGenbank2.sqn PTU10 KM053618

TaureatrnGtrnSGenbank2.sqn PTU11 KM053619

TaureatrnGtrnSGenbank2.sqn PTU12 KM053620

TaureatrnGtrnSGenbank2.sqn PTU13 KM053621

TaureatrnGtrnSGenbank2.sqn PTU14 KM053622

TaureatrnGtrnSGenbank2.sqn PTU15 KM053623

TaureatrnGtrnSGenbank2.sqn PTU16 KM053624

TaureatrnGtrnSGenbank2.sqn PTU18 KM053625

TaureatrnGtrnSGenbank2.sqn SAF01 KM053626

TaureatrnGtrnSGenbank2.sqn SAF02 KM053627

TaureatrnGtrnSGenbank2.sqn SAF03 KM053628

TaureatrnGtrnSGenbank2.sqn SAF04 KM053629

TaureatrnGtrnSGenbank2.sqn SAF06 KM053630

TaureatrnGtrnSGenbank2.sqn SAF05 KM053631

TaureatrnGtrnSGenbank2.sqn SAF07 KM053632

TaureatrnGtrnSGenbank2.sqn SAF08 KM053633

TaureatrnGtrnSGenbank2.sqn SAF09 KM053634

TaureatrnGtrnSGenbank2.sqn SAF10 KM053635

TaureatrnGtrnSGenbank2.sqn SAF11 KM053636

TaureatrnGtrnSGenbank2.sqn SAF12 KM053637

TaureatrnGtrnSGenbank2.sqn SAF13 KM053638

TaureatrnGtrnSGenbank2.sqn SAF14 KM053639

TaureatrnGtrnSGenbank2.sqn SAF15 KM053640

TaureatrnGtrnSGenbank2.sqn SAF16 KM053641

TaureatrnGtrnSGenbank2.sqn SCA01 KM053642

TaureatrnGtrnSGenbank2.sqn SCA02 KM053643

TaureatrnGtrnSGenbank2.sqn SCA03 KM053644

TaureatrnGtrnSGenbank2.sqn SCA04 KM053645

TaureatrnGtrnSGenbank2.sqn SCA05 KM053646

TaureatrnGtrnSGenbank2.sqn SCA06 KM053647

TaureatrnGtrnSGenbank2.sqn SCA07 KM053648

TaureatrnGtrnSGenbank2.sqn SCA09 KM053649

TaureatrnGtrnSGenbank2.sqn SCA10 KM053650

TaureatrnGtrnSGenbank2.sqn SCA11 KM053651

TaureatrnGtrnSGenbank2.sqn SCA12 KM053652

TaureatrnGtrnSGenbank2.sqn SCA14 KM053653

TaureatrnGtrnSGenbank2.sqn SCA15 KM053654

TaureatrnGtrnSGenbank2.sqn SCA16 KM053655

TaureatrnGtrnSGenbank2.sqn SCA17 KM053656

TaureatrnGtrnSGenbank2.sqn SDO01 KM053657

TaureatrnGtrnSGenbank2.sqn SDO02 KM053658

TaureatrnGtrnSGenbank2.sqn SDO03 KM053659

TaureatrnGtrnSGenbank2.sqn SEC01 KM053660

TaureatrnGtrnSGenbank2.sqn SEC02 KM053661

TaureatrnGtrnSGenbank2.sqn SEC03 KM053662

TaureatrnGtrnSGenbank2.sqn SEC04 KM053663

TaureatrnGtrnSGenbank2.sqn STZ01 KM053664

TaureatrnGtrnSGenbank2.sqn STZ02 KM053665

TaureatrnGtrnSGenbank2.sqn STZ03 KM053666

TaureatrnGtrnSGenbank2.sqn STZ04 KM053667

TaureatrnGtrnSGenbank2.sqn STZ05 KM053668

TaureatrnGtrnSGenbank2.sqn STZ06 KM053669

TaureatrnGtrnSGenbank2.sqn STZ07 KM053670

TaureatrnGtrnSGenbank2.sqn STZ08 KM053671

TaureatrnGtrnSGenbank2.sqn STZ09 KM053672

TaureatrnGtrnSGenbank2.sqn STZ10 KM053673

TaureatrnGtrnSGenbank2.sqn STZ11 KM053674

TaureatrnGtrnSGenbank2.sqn STZ12 KM053675

TaureatrnGtrnSGenbank2.sqn STZ13 KM053676

TaureatrnGtrnSGenbank2.sqn STZ14 KM053677

TaureatrnGtrnSGenbank2.sqn STZ15 KM053678

TaureatrnGtrnSGenbank2.sqn STZ16 KM053679

TaureatrnGtrnSGenbank2.sqn STZ17 KM053680

TaureatrnGtrnSGenbank2.sqn SUM01 KM053681

TaureatrnGtrnSGenbank2.sqn SUM02 KM053682

TaureatrnGtrnSGenbank2.sqn SUM03 KM053683

TaureatrnGtrnSGenbank2.sqn SUM04 KM053684

TaureatrnGtrnSGenbank2.sqn SUM05 KM053685

TaureatrnGtrnSGenbank2.sqn SUM06 KM053686

TaureatrnGtrnSGenbank2.sqn SUM07 KM053687

TaureatrnGtrnSGenbank2.sqn SUM08 KM053688

TaureatrnGtrnSGenbank2.sqn SUM09 KM053689

TaureatrnGtrnSGenbank2.sqn SUM10 KM053690

TaureatrnGtrnSGenbank2.sqn SUM11 KM053691

TaureatrnGtrnSGenbank2.sqn SUM12 KM053692

TaureatrnGtrnSGenbank2.sqn SUM13 KM053693

TaureatrnGtrnSGenbank2.sqn SUM14 KM053694

TaureatrnGtrnSGenbank2.sqn VIB01 KM053695

TaureatrnGtrnSGenbank2.sqn VIB02 KM053696

TaureatrnGtrnSGenbank2.sqn VIB03 KM053697

TaureatrnGtrnSGenbank2.sqn VIB04 KM053698

TaureatrnGtrnSGenbank2.sqn VIB05 KM053699

TaureatrnGtrnSGenbank2.sqn VIB06 KM053700

TaureatrnGtrnSGenbank2.sqn VIB07 KM053701

TaureatrnGtrnSGenbank2.sqn VIB08 KM053702

TaureatrnGtrnSGenbank2.sqn VIB09 KM053703

TaureatrnGtrnSGenbank2.sqn VIB10 KM053704

TaureatrnGtrnSGenbank2.sqn VIB11 KM053705

TaureatrnGtrnSGenbank2.sqn VIB12 KM053706

TaureatrnGtrnSGenbank2.sqn VIB13 KM053707

TaureatrnGtrnSGenbank2.sqn VIB14 KM053708

TaureatrnGtrnSGenbank2.sqn VIB15 KM053709

TaureatrnGtrnSGenbank2.sqn VIB16 KM053710

Taureaycf6Genbank2.sqn AGE03 KM053711

Taureaycf6Genbank2.sqn AGE06 KM053712

Taureaycf6Genbank2.sqn AGE08 KM053713

Taureaycf6Genbank2.sqn AGE13 KM053714

Taureaycf6Genbank2.sqn AGE17 KM053715

Taureaycf6Genbank2.sqn AGE18 KM053716

Taureaycf6Genbank2.sqn AGE19 KM053717

Taureaycf6Genbank2.sqn AGE20 KM053718

Taureaycf6Genbank2.sqn AGE23 KM053719

Taureaycf6Genbank2.sqn AGE28 KM053720

Taureaycf6Genbank2.sqn AGE30 KM053721

Taureaycf6Genbank2.sqn AGE31 KM053722

Taureaycf6Genbank2.sqn AGE32 KM053723

Taureaycf6Genbank2.sqn AGE33 KM053724

Taureaycf6Genbank2.sqn AGE35 KM053725

Taureaycf6Genbank2.sqn AGE36 KM053726

Taureaycf6Genbank2.sqn BAG04 KM053727

Taureaycf6Genbank2.sqn ARA01 KM053728

Taureaycf6Genbank2.sqn ARA02 KM053729

Taureaycf6Genbank2.sqn ARA04 KM053730

Taureaycf6Genbank2.sqn ARA05 KM053731

Taureaycf6Genbank2.sqn ARA06 KM053732

Taureaycf6Genbank2.sqn ARA07 KM053733

Taureaycf6Genbank2.sqn ARA08 KM053734

Taureaycf6Genbank2.sqn ARA09 KM053735

Taureaycf6Genbank2.sqn ARA10 KM053736

Taureaycf6Genbank2.sqn ARA11 KM053737

Taureaycf6Genbank2.sqn ARA12 KM053738

Taureaycf6Genbank2.sqn ARA13 KM053739

Taureaycf6Genbank2.sqn ARA14 KM053740

Taureaycf6Genbank2.sqn BAG01 KM053741

Taureaycf6Genbank2.sqn BAG02 KM053742

Taureaycf6Genbank2.sqn BAG034 KM053743

Taureaycf6Genbank2.sqn BAG05 KM053744

Taureaycf6Genbank2.sqn BAG07 KM053745

Taureaycf6Genbank2.sqn BAG08 KM053746

Taureaycf6Genbank2.sqn BAG09 KM053747

Taureaycf6Genbank2.sqn BAG10 KM053748

Taureaycf6Genbank2.sqn BAG11 KM053749

Taureaycf6Genbank2.sqn BAG13 KM053750

Taureaycf6Genbank2.sqn BAG15 KM053751

Taureaycf6Genbank2.sqn BAG16 KM053752

Taureaycf6Genbank2.sqn BAG17 KM053753

Taureaycf6Genbank2.sqn BAG18 KM053754

Taureaycf6Genbank2.sqn BAG19 KM053755

Taureaycf6Genbank2.sqn BAG20 KM053756

Taureaycf6Genbank2.sqn BAG21 KM053757

Taureaycf6Genbank2.sqn BAG27 KM053758

Taureaycf6Genbank2.sqn BAR01 KM053759

Taureaycf6Genbank2.sqn BAR02 KM053760

Taureaycf6Genbank2.sqn BAR03 KM053761

Taureaycf6Genbank2.sqn BAR04 KM053762

Taureaycf6Genbank2.sqn BAR05 KM053763

Taureaycf6Genbank2.sqn BAR09 KM053764

Taureaycf6Genbank2.sqn BAR10 KM053765

Taureaycf6Genbank2.sqn BAR11 KM053766

Taureaycf6Genbank2.sqn BAR12 KM053767

Taureaycf6Genbank2.sqn BAR13 KM053768

Taureaycf6Genbank2.sqn BAR15 KM053769

Taureaycf6Genbank2.sqn BAR16 KM053770

Taureaycf6Genbank2.sqn BOD01 KM053771

Taureaycf6Genbank2.sqn BOD02 KM053772

Taureaycf6Genbank2.sqn BOD03 KM053773

Taureaycf6Genbank2.sqn BOD06 KM053774

Taureaycf6Genbank2.sqn BOD07 KM053775

Taureaycf6Genbank2.sqn BOD08 KM053776

Taureaycf6Genbank2.sqn BOD09 KM053777

Taureaycf6Genbank2.sqn BOD10 KM053778

Taureaycf6Genbank2.sqn BOD11 KM053779

Taureaycf6Genbank2.sqn BOD12 KM053780

Taureaycf6Genbank2.sqn BOD14 KM053781

Taureaycf6Genbank2.sqn BOD15 KM053782

Taureaycf6Genbank2.sqn BOD16 KM053783

Taureaycf6Genbank2.sqn BOD17 KM053784

Taureaycf6Genbank2.sqn BOD18 KM053785

Taureaycf6Genbank2.sqn BOD19 KM053786

Taureaycf6Genbank2.sqn CAR01 KM053787

Taureaycf6Genbank2.sqn CAR02 KM053788

Taureaycf6Genbank2.sqn CAR03 KM053789

Taureaycf6Genbank2.sqn CAR04 KM053790

Taureaycf6Genbank2.sqn CAR05 KM053791

Taureaycf6Genbank2.sqn CAR06 KM053792

Taureaycf6Genbank2.sqn CAR07 KM053793

Taureaycf6Genbank2.sqn CAR08 KM053794

Taureaycf6Genbank2.sqn CAR09 KM053795

Taureaycf6Genbank2.sqn CAR10 KM053796

Taureaycf6Genbank2.sqn CAR11 KM053797

Taureaycf6Genbank2.sqn CAR12 KM053798

Taureaycf6Genbank2.sqn CAR13 KM053799

Taureaycf6Genbank2.sqn CAR14 KM053800

Taureaycf6Genbank2.sqn CAR15 KM053801

Taureaycf6Genbank2.sqn CAR16 KM053802

Taureaycf6Genbank2.sqn CHG01 KM053803

Taureaycf6Genbank2.sqn CHG02 KM053804

Taureaycf6Genbank2.sqn CHG03 KM053805

Taureaycf6Genbank2.sqn CHG04 KM053806

Taureaycf6Genbank2.sqn CHG05 KM053807

Taureaycf6Genbank2.sqn CHG06 KM053808

Taureaycf6Genbank2.sqn CHG07 KM053809

Taureaycf6Genbank2.sqn CHG09 KM053810

Taureaycf6Genbank2.sqn CHG10 KM053811

Taureaycf6Genbank2.sqn CHG11 KM053812

Taureaycf6Genbank2.sqn CHG12 KM053813

Taureaycf6Genbank2.sqn CHG13 KM053814

Taureaycf6Genbank2.sqn CHG14 KM053815

Taureaycf6Genbank2.sqn CHG15 KM053816

Taureaycf6Genbank2.sqn CHG16 KM053817

Taureaycf6Genbank2.sqn FAT01 KM053818

Taureaycf6Genbank2.sqn FAT02 KM053819

Taureaycf6Genbank2.sqn FAT03 KM053820

Taureaycf6Genbank2.sqn FAT04 KM053821

Taureaycf6Genbank2.sqn FAT05 KM053822

Taureaycf6Genbank2.sqn FAT06 KM053823

Taureaycf6Genbank2.sqn FAT11 KM053824

Taureaycf6Genbank2.sqn FAT13 KM053825

Taureaycf6Genbank2.sqn FAT14 KM053826

Taureaycf6Genbank2.sqn FAT15 KM053827

Taureaycf6Genbank2.sqn FAT16 KM053828

Taureaycf6Genbank2.sqn FAT20 KM053829

Taureaycf6Genbank2.sqn FAT21 KM053830

Taureaycf6Genbank2.sqn FAT22 KM053831

Taureaycf6Genbank2.sqn FAT23 KM053832

Taureaycf6Genbank2.sqn FAT31 KM053833

Taureaycf6Genbank2.sqn FAT33 KM053834

Taureaycf6Genbank2.sqn FAT35 KM053835

Taureaycf6Genbank2.sqn FAT36 KM053836

Taureaycf6Genbank2.sqn FAT37 KM053837

Taureaycf6Genbank2.sqn FAT38 KM053838

Taureaycf6Genbank2.sqn GSV01 KM053839

Taureaycf6Genbank2.sqn GSV02 KM053840

Taureaycf6Genbank2.sqn GSV03 KM053841

Taureaycf6Genbank2.sqn GSV04 KM053842

Taureaycf6Genbank2.sqn GSV05 KM053843

Taureaycf6Genbank2.sqn GSV06 KM053844

Taureaycf6Genbank2.sqn GSV07 KM053845

Taureaycf6Genbank2.sqn GSV08 KM053846

Taureaycf6Genbank2.sqn NIQ01 KM053847

Taureaycf6Genbank2.sqn NIQ02 KM053848

Taureaycf6Genbank2.sqn NIQ03 KM053849

Taureaycf6Genbank2.sqn NIQ04 KM053850

Taureaycf6Genbank2.sqn NIQ05 KM053851

Taureaycf6Genbank2.sqn NIQ06 KM053852

Taureaycf6Genbank2.sqn NIQ08 KM053853

Taureaycf6Genbank2.sqn NIQ10 KM053854

Taureaycf6Genbank2.sqn NIQ14 KM053855

Taureaycf6Genbank2.sqn NIQ17 KM053856

Taureaycf6Genbank2.sqn NIQ19 KM053857

Taureaycf6Genbank2.sqn NIQ22 KM053858

Taureaycf6Genbank2.sqn NIQ25 KM053859

Taureaycf6Genbank2.sqn NIQ26 KM053860

Taureaycf6Genbank2.sqn PAN01 KM053861

Taureaycf6Genbank2.sqn PAN02 KM053862

Taureaycf6Genbank2.sqn PAN03 KM053863

Taureaycf6Genbank2.sqn PAN04 KM053864

Taureaycf6Genbank2.sqn PAN05 KM053865

Taureaycf6Genbank2.sqn PAN06 KM053866

Taureaycf6Genbank2.sqn PAN07 KM053867

Taureaycf6Genbank2.sqn PAN08 KM053868

Taureaycf6Genbank2.sqn PAN09 KM053869

Taureaycf6Genbank2.sqn PAN10 KM053870

Taureaycf6Genbank2.sqn PAN11 KM053871

Taureaycf6Genbank2.sqn PAN12 KM053872

Taureaycf6Genbank2.sqn PAN13 KM053873

Taureaycf6Genbank2.sqn PAN14 KM053874

Taureaycf6Genbank2.sqn PAN15 KM053875

Taureaycf6Genbank2.sqn PAN16 KM053876

Taureaycf6Genbank2.sqn PNE01 KM053877

Taureaycf6Genbank2.sqn PNE02 KM053878

Taureaycf6Genbank2.sqn PNE03 KM053879

Taureaycf6Genbank2.sqn PNE04 KM053880

Taureaycf6Genbank2.sqn PNE05 KM053881

Taureaycf6Genbank2.sqn PNE06 KM053882

Taureaycf6Genbank2.sqn PNE07 KM053883

Taureaycf6Genbank2.sqn PNE08 KM053884

Taureaycf6Genbank2.sqn PNE09 KM053885

Taureaycf6Genbank2.sqn PNE10 KM053886

Taureaycf6Genbank2.sqn PNE11 KM053887

Taureaycf6Genbank2.sqn PNE12 KM053888

Taureaycf6Genbank2.sqn PNE13 KM053889

Taureaycf6Genbank2.sqn PNE14 KM053890

Taureaycf6Genbank2.sqn PNE15 KM053891

Taureaycf6Genbank2.sqn PNE16 KM053892

Taureaycf6Genbank2.sqn POT01 KM053893

Taureaycf6Genbank2.sqn POT02 KM053894

Taureaycf6Genbank2.sqn POT03 KM053895

Taureaycf6Genbank2.sqn POT04 KM053896

Taureaycf6Genbank2.sqn POT05 KM053897

Taureaycf6Genbank2.sqn POT06 KM053898

Taureaycf6Genbank2.sqn POT07 KM053899

Taureaycf6Genbank2.sqn POT08 KM053900

Taureaycf6Genbank2.sqn POT09 KM053901

Taureaycf6Genbank2.sqn POT10 KM053902

Taureaycf6Genbank2.sqn POT11 KM053903

Taureaycf6Genbank2.sqn POT12 KM053904

Taureaycf6Genbank2.sqn POT13 KM053905

Taureaycf6Genbank2.sqn POT14 KM053906

Taureaycf6Genbank2.sqn POT15 KM053907

Taureaycf6Genbank2.sqn POT16 KM053908

Taureaycf6Genbank2.sqn PTU01 KM053909

Taureaycf6Genbank2.sqn PTU02 KM053910

Taureaycf6Genbank2.sqn PTU03 KM053911

Taureaycf6Genbank2.sqn PTU04 KM053912

Taureaycf6Genbank2.sqn PTU05 KM053913

Taureaycf6Genbank2.sqn PTU06 KM053914

Taureaycf6Genbank2.sqn PTU07 KM053915

Taureaycf6Genbank2.sqn PTU09 KM053916

Taureaycf6Genbank2.sqn PTU10 KM053917

Taureaycf6Genbank2.sqn PTU11 KM053918

Taureaycf6Genbank2.sqn PTU12 KM053919

Taureaycf6Genbank2.sqn PTU13 KM053920

Taureaycf6Genbank2.sqn PTU14 KM053921

Taureaycf6Genbank2.sqn PTU15 KM053922

Taureaycf6Genbank2.sqn PTU16 KM053923

Taureaycf6Genbank2.sqn PTU18 KM053924

Taureaycf6Genbank2.sqn SAF01 KM053925

Taureaycf6Genbank2.sqn SAF02 KM053926

Taureaycf6Genbank2.sqn SAF03 KM053927

Taureaycf6Genbank2.sqn SAF04 KM053928

Taureaycf6Genbank2.sqn SAF05 KM053929

Taureaycf6Genbank2.sqn SAF06 KM053930

Taureaycf6Genbank2.sqn SAF07 KM053931

Taureaycf6Genbank2.sqn SAF08 KM053932

Taureaycf6Genbank2.sqn SAF09 KM053933

Taureaycf6Genbank2.sqn SAF10 KM053934

Taureaycf6Genbank2.sqn SAF11 KM053935

Taureaycf6Genbank2.sqn SAF12 KM053936

Taureaycf6Genbank2.sqn SAF13 KM053937

Taureaycf6Genbank2.sqn SAF14 KM053938

Taureaycf6Genbank2.sqn SAF15 KM053939

Taureaycf6Genbank2.sqn SAF16 KM053940

Taureaycf6Genbank2.sqn SCA01 KM053941

Taureaycf6Genbank2.sqn SCA02 KM053942

Taureaycf6Genbank2.sqn SCA03 KM053943

Taureaycf6Genbank2.sqn SCA04 KM053944

Taureaycf6Genbank2.sqn SCA05 KM053945

Taureaycf6Genbank2.sqn SCA06 KM053946

Taureaycf6Genbank2.sqn SCA07 KM053947

Taureaycf6Genbank2.sqn SCA09 KM053948

Taureaycf6Genbank2.sqn SCA10 KM053949

Taureaycf6Genbank2.sqn SCA11 KM053950

Taureaycf6Genbank2.sqn SCA12 KM053951

Taureaycf6Genbank2.sqn SCA14 KM053952

Taureaycf6Genbank2.sqn SCA15 KM053953

Taureaycf6Genbank2.sqn SCA16 KM053954

Taureaycf6Genbank2.sqn SCA17 KM053955

Taureaycf6Genbank2.sqn SDO01 KM053956

Taureaycf6Genbank2.sqn SDO02 KM053957

Taureaycf6Genbank2.sqn SDO03 KM053958

Taureaycf6Genbank2.sqn SEC01 KM053959

Taureaycf6Genbank2.sqn SEC02 KM053960

Taureaycf6Genbank2.sqn SEC03 KM053961

Taureaycf6Genbank2.sqn SEC04 KM053962

Taureaycf6Genbank2.sqn STZ01 KM053963

Taureaycf6Genbank2.sqn STZ02 KM053964

Taureaycf6Genbank2.sqn STZ03 KM053965

Taureaycf6Genbank2.sqn STZ04 KM053966

Taureaycf6Genbank2.sqn STZ05 KM053967

Taureaycf6Genbank2.sqn STZ06 KM053968

Taureaycf6Genbank2.sqn STZ07 KM053969

Taureaycf6Genbank2.sqn STZ08 KM053970

Taureaycf6Genbank2.sqn STZ09 KM053971

Taureaycf6Genbank2.sqn STZ10 KM053972

Taureaycf6Genbank2.sqn STZ11 KM053973

Taureaycf6Genbank2.sqn STZ12 KM053974

Taureaycf6Genbank2.sqn STZ13 KM053975

Taureaycf6Genbank2.sqn STZ14 KM053976

Taureaycf6Genbank2.sqn STZ15 KM053977

Taureaycf6Genbank2.sqn STZ16 KM053978

Taureaycf6Genbank2.sqn STZ17 KM053979

Taureaycf6Genbank2.sqn SUM01 KM053980

Taureaycf6Genbank2.sqn SUM02 KM053981

Taureaycf6Genbank2.sqn SUM03 KM053982

Taureaycf6Genbank2.sqn SUM04 KM053983

Taureaycf6Genbank2.sqn SUM05 KM053984

Taureaycf6Genbank2.sqn SUM06 KM053985

Taureaycf6Genbank2.sqn SUM07 KM053986

Taureaycf6Genbank2.sqn SUM08 KM053987

Taureaycf6Genbank2.sqn SUM09 KM053988

Taureaycf6Genbank2.sqn SUM10 KM053989

Taureaycf6Genbank2.sqn SUM11 KM053990

Taureaycf6Genbank2.sqn SUM12 KM053991

Taureaycf6Genbank2.sqn SUM13 KM053992

Taureaycf6Genbank2.sqn SUM14 KM053993

Taureaycf6Genbank2.sqn VIB01 KM053994

Taureaycf6Genbank2.sqn VIB02 KM053995

Taureaycf6Genbank2.sqn VIB03 KM053996

Taureaycf6Genbank2.sqn VIB04 KM053997

Taureaycf6Genbank2.sqn VIB05 KM053998

Taureaycf6Genbank2.sqn VIB06 KM053999

Taureaycf6Genbank2.sqn VIB07 KM054000

Taureaycf6Genbank2.sqn VIB08 KM054001

Taureaycf6Genbank2.sqn VIB09 KM054002

Taureaycf6Genbank2.sqn VIB10 KM054003

Taureaycf6Genbank2.sqn VIB11 KM054004

Taureaycf6Genbank2.sqn VIB12 KM054005

Taureaycf6Genbank2.sqn VIB13 KM054006

Taureaycf6Genbank2.sqn VIB14 KM054007

Taureaycf6Genbank2.sqn VIB15 KM054008

Taureaycf6Genbank2.sqn VIB16 KM054009

TaureaITSGenBank4.sqn AGE03 KM054010

TaureaITSGenBank4.sqn AGE06 KM054011

TaureaITSGenBank4.sqn AGE13 KM054012

TaureaITSGenBank4.sqn AGE17 KM054013

TaureaITSGenBank4.sqn AGE18 KM054014

TaureaITSGenBank4.sqn AGE19 KM054015

TaureaITSGenBank4.sqn AGE20 KM054016

TaureaITSGenBank4.sqn AGE23 KM054017

TaureaITSGenBank4.sqn AGE28 KM054018

TaureaITSGenBank4.sqn AGE30 KM054019

TaureaITSGenBank4.sqn AGE32 KM054020

TaureaITSGenBank4.sqn AGE33 KM054021

TaureaITSGenBank4.sqn AGE35 KM054022

TaureaITSGenBank4.sqn AGE36 KM054023

TaureaITSGenBank4.sqn ARA01 KM054024

TaureaITSGenBank4.sqn ARA02 KM054025

TaureaITSGenBank4.sqn ARA03 KM054026

TaureaITSGenBank4.sqn ARA04 KM054027

TaureaITSGenBank4.sqn ARA05 KM054028

TaureaITSGenBank4.sqn ARA06 KM054029

TaureaITSGenBank4.sqn ARA07 KM054030

TaureaITSGenBank4.sqn ARA08 KM054031

TaureaITSGenBank4.sqn ARA09 KM054032

TaureaITSGenBank4.sqn ARA10 KM054033

TaureaITSGenBank4.sqn ARA11 KM054034

TaureaITSGenBank4.sqn ARA12 KM054035

TaureaITSGenBank4.sqn ARA13 KM054036

TaureaITSGenBank4.sqn ARA14 KM054037

TaureaITSGenBank4.sqn BAG02 KM054038

TaureaITSGenBank4.sqn BAG04 KM054039

TaureaITSGenBank4.sqn BAG05 KM054040

TaureaITSGenBank4.sqn BAG07 KM054041

TaureaITSGenBank4.sqn BAG08 KM054042

TaureaITSGenBank4.sqn BAG09 KM054043

TaureaITSGenBank4.sqn BAG10 KM054044

TaureaITSGenBank4.sqn BAG13 KM054045

TaureaITSGenBank4.sqn BAG15 KM054046

TaureaITSGenBank4.sqn BAG18 KM054047

TaureaITSGenBank4.sqn BAG19 KM054048

TaureaITSGenBank4.sqn BAG20 KM054049

TaureaITSGenBank4.sqn BAR03 KM054050

TaureaITSGenBank4.sqn BAR04 KM054051

TaureaITSGenBank4.sqn BAR05 KM054052

TaureaITSGenBank4.sqn BAR09 KM054053

TaureaITSGenBank4.sqn BAR10 KM054054

TaureaITSGenBank4.sqn BAR11 KM054055

TaureaITSGenBank4.sqn BAR12 KM054056

TaureaITSGenBank4.sqn BAR13 KM054057

TaureaITSGenBank4.sqn BAR16 KM054058

TaureaITSGenBank4.sqn BOD02 KM054059

TaureaITSGenBank4.sqn BOD03 KM054060

TaureaITSGenBank4.sqn BOD06 KM054061

TaureaITSGenBank4.sqn BOD07 KM054062

TaureaITSGenBank4.sqn BOD08 KM054063

TaureaITSGenBank4.sqn BOD09 KM054064

TaureaITSGenBank4.sqn BOD11 KM054065

TaureaITSGenBank4.sqn BOD12 KM054066

TaureaITSGenBank4.sqn BOD13 KM054067

TaureaITSGenBank4.sqn BOD14 KM054068

TaureaITSGenBank4.sqn BOD15 KM054069

TaureaITSGenBank4.sqn BOD16 KM054070

TaureaITSGenBank4.sqn CAR01 KM054071

TaureaITSGenBank4.sqn CAR04 KM054072

TaureaITSGenBank4.sqn CAR06 KM054073

TaureaITSGenBank4.sqn CAR10 KM054074

TaureaITSGenBank4.sqn CAR11 KM054075

TaureaITSGenBank4.sqn CAR12 KM054076

TaureaITSGenBank4.sqn CAR13 KM054077

TaureaITSGenBank4.sqn CAR15 KM054078

TaureaITSGenBank4.sqn CAR16 KM054079

TaureaITSGenBank4.sqn CHG03 KM054080

TaureaITSGenBank4.sqn CHG04 KM054081

TaureaITSGenBank4.sqn CHG05 KM054082

TaureaITSGenBank4.sqn CHG06 KM054083

TaureaITSGenBank4.sqn CHG07 KM054084

TaureaITSGenBank4.sqn CHG09 KM054085

TaureaITSGenBank4.sqn CHG10 KM054086

TaureaITSGenBank4.sqn CHG12 KM054087

TaureaITSGenBank4.sqn CHG13 KM054088

TaureaITSGenBank4.sqn CHG14 KM054089

TaureaITSGenBank4.sqn CHG16 KM054090

TaureaITSGenBank4.sqn FAT02 KM054091

TaureaITSGenBank4.sqn FAT04 KM054092

TaureaITSGenBank4.sqn FAT05 KM054093

TaureaITSGenBank4.sqn FAT07 KM054094

TaureaITSGenBank4.sqn FAT10 KM054095

TaureaITSGenBank4.sqn FAT12 KM054096

TaureaITSGenBank4.sqn FAT14 KM054097

TaureaITSGenBank4.sqn FAT15 KM054098

TaureaITSGenBank4.sqn FAT21 KM054099

TaureaITSGenBank4.sqn FAT22 KM054100

TaureaITSGenBank4.sqn FAT31 KM054101

TaureaITSGenBank4.sqn FAT36 KM054102

TaureaITSGenBank4.sqn FAT37 KM054103

TaureaITSGenBank4.sqn FAT38 KM054104

TaureaITSGenBank4.sqn GSV01 KM054105

TaureaITSGenBank4.sqn GSV02 KM054106

TaureaITSGenBank4.sqn GSV04 KM054107

TaureaITSGenBank4.sqn GSV05 KM054108

TaureaITSGenBank4.sqn GSV07 KM054109

TaureaITSGenBank4.sqn NIQ01 KM054110

TaureaITSGenBank4.sqn NIQ02 KM054111

TaureaITSGenBank4.sqn NIQ03 KM054112

TaureaITSGenBank4.sqn NIQ05 KM054113

TaureaITSGenBank4.sqn NIQ08 KM054114

TaureaITSGenBank4.sqn NIQ14 KM054115

TaureaITSGenBank4.sqn NIQ17 KM054116

TaureaITSGenBank4.sqn PAN01 KM054117

TaureaITSGenBank4.sqn PAN02 KM054118

TaureaITSGenBank4.sqn PAN03 KM054119

TaureaITSGenBank4.sqn PAN04 KM054120

TaureaITSGenBank4.sqn PAN05 KM054121

TaureaITSGenBank4.sqn PAN06 KM054122

TaureaITSGenBank4.sqn PAN07 KM054123

TaureaITSGenBank4.sqn PAN09 KM054124

TaureaITSGenBank4.sqn PAN10 KM054125

TaureaITSGenBank4.sqn PAN11 KM054126

TaureaITSGenBank4.sqn PAN12 KM054127

TaureaITSGenBank4.sqn PAN13 KM054128

TaureaITSGenBank4.sqn PAN14 KM054129

TaureaITSGenBank4.sqn PAN15 KM054130

TaureaITSGenBank4.sqn PAN16 KM054131

TaureaITSGenBank4.sqn PNE01 KM054132

TaureaITSGenBank4.sqn PNE02 KM054133

TaureaITSGenBank4.sqn PNE03 KM054134

TaureaITSGenBank4.sqn PNE04 KM054135

TaureaITSGenBank4.sqn PNE05 KM054136

TaureaITSGenBank4.sqn PNE06 KM054137

TaureaITSGenBank4.sqn PNE07 KM054138

TaureaITSGenBank4.sqn PNE08 KM054139

TaureaITSGenBank4.sqn PNE09 KM054140

TaureaITSGenBank4.sqn PNE10 KM054141

TaureaITSGenBank4.sqn PNE11 KM054142

TaureaITSGenBank4.sqn PNE12 KM054143

TaureaITSGenBank4.sqn PNE13 KM054144

TaureaITSGenBank4.sqn PNE14 KM054145

TaureaITSGenBank4.sqn PNE15 KM054146

TaureaITSGenBank4.sqn PNE16 KM054147

TaureaITSGenBank4.sqn POT01 KM054148

TaureaITSGenBank4.sqn POT02 KM054149

TaureaITSGenBank4.sqn POT03 KM054150

TaureaITSGenBank4.sqn POT04 KM054151

TaureaITSGenBank4.sqn POT05 KM054152

TaureaITSGenBank4.sqn POT06 KM054153

TaureaITSGenBank4.sqn POT07 KM054154

TaureaITSGenBank4.sqn POT08 KM054155

TaureaITSGenBank4.sqn POT09 KM054156

TaureaITSGenBank4.sqn POT10 KM054157

TaureaITSGenBank4.sqn POT11 KM054158

TaureaITSGenBank4.sqn POT12 KM054159

TaureaITSGenBank4.sqn POT13 KM054160

TaureaITSGenBank4.sqn POT14 KM054161

TaureaITSGenBank4.sqn POT15 KM054162

TaureaITSGenBank4.sqn POT16 KM054163

TaureaITSGenBank4.sqn SAF04 KM054164

TaureaITSGenBank4.sqn SAF05 KM054165

TaureaITSGenBank4.sqn SAF07 KM054166

TaureaITSGenBank4.sqn SAF02 KM054167

TaureaITSGenBank4.sqn SAF15 KM054168

TaureaITSGenBank4.sqn SAF01 KM054169

TaureaITSGenBank4.sqn SAF16 KM054170

TaureaITSGenBank4.sqn SCA06 KM054171

TaureaITSGenBank4.sqn SCA09 KM054172

TaureaITSGenBank4.sqn SCA10 KM054173

TaureaITSGenBank4.sqn SCA11 KM054174

TaureaITSGenBank4.sqn SCA12 KM054175

TaureaITSGenBank4.sqn SCA13 KM054176

TaureaITSGenBank4.sqn SCA16 KM054177

TaureaITSGenBank4.sqn SDO02 KM054178

TaureaITSGenBank4.sqn SEC02 KM054179

TaureaITSGenBank4.sqn SEC03 KM054180

TaureaITSGenBank4.sqn SEC04 KM054181

TaureaITSGenBank4.sqn STZ02 KM054182

TaureaITSGenBank4.sqn STZ07 KM054183

TaureaITSGenBank4.sqn STZ08 KM054184

TaureaITSGenBank4.sqn STZ09 KM054185

TaureaITSGenBank4.sqn STZ10 KM054186

TaureaITSGenBank4.sqn STZ11 KM054187

TaureaITSGenBank4.sqn STZ12 KM054188

TaureaITSGenBank4.sqn STZ13 KM054189

TaureaITSGenBank4.sqn STZ18 KM054190

TaureaITSGenBank4.sqn SUM01 KM054191

TaureaITSGenBank4.sqn SUM02 KM054192

TaureaITSGenBank4.sqn SUM03 KM054193

TaureaITSGenBank4.sqn SUM04 KM054194

TaureaITSGenBank4.sqn SUM05 KM054195

TaureaITSGenBank4.sqn SUM06 KM054196

TaureaITSGenBank4.sqn SUM08 KM054197

TaureaITSGenBank4.sqn SUM07 KM054198

TaureaITSGenBank4.sqn SUM09 KM054199

TaureaITSGenBank4.sqn SUM11 KM054200

TaureaITSGenBank4.sqn SUM13 KM054201

TaureaITSGenBank4.sqn SUM14 KM054202

TaureaITSGenBank4.sqn VIB02 KM054203

TaureaITSGenBank4.sqn VIB03 KM054204

TaureaITSGenBank4.sqn VIB04 KM054205

TaureaITSGenBank4.sqn VIB05 KM054206

TaureaITSGenBank4.sqn VIB06 KM054207

TaureaITSGenBank4.sqn VIB07 KM054208

TaureaITSGenBank4.sqn VIB08 KM054209

TaureaITSGenBank4.sqn VIB09 KM054210

TaureaITSGenBank4.sqn VIB10 KM054211

TaureaITSGenBank4.sqn VIB12 KM054212

TaureaITSGenBank4.sqn VIB13 KM054213

TaureaITSGenBank4.sqn VIB16 KM054214

TaupsbAGenBank4.sqn AGE03 KM054215

TaupsbAGenBank4.sqn AGE06 KM054216

TaupsbAGenBank4.sqn AGE08 KM054217

TaupsbAGenBank4.sqn AGE13 KM054218

TaupsbAGenBank4.sqn AGE17 KM054219

TaupsbAGenBank4.sqn AGE18 KM054220

TaupsbAGenBank4.sqn AGE19 KM054221

TaupsbAGenBank4.sqn AGE20 KM054222

TaupsbAGenBank4.sqn AGE23 KM054223

TaupsbAGenBank4.sqn AGE28 KM054224

TaupsbAGenBank4.sqn AGE30 KM054225

TaupsbAGenBank4.sqn AGE31 KM054226

TaupsbAGenBank4.sqn AGE32 KM054227

TaupsbAGenBank4.sqn AGE33 KM054228

TaupsbAGenBank4.sqn AGE35 KM054229

TaupsbAGenBank4.sqn AGE36 KM054230

TaupsbAGenBank4.sqn ARA01 KM054231

TaupsbAGenBank4.sqn ARA02 KM054232

TaupsbAGenBank4.sqn ARA04 KM054233

TaupsbAGenBank4.sqn ARA05 KM054234

TaupsbAGenBank4.sqn ARA06 KM054235

TaupsbAGenBank4.sqn ARA07 KM054236

TaupsbAGenBank4.sqn ARA08 KM054237

TaupsbAGenBank4.sqn ARA09 KM054238

TaupsbAGenBank4.sqn ARA10 KM054239

TaupsbAGenBank4.sqn ARA11 KM054240

TaupsbAGenBank4.sqn ARA12 KM054241

TaupsbAGenBank4.sqn ARA13 KM054242

TaupsbAGenBank4.sqn ARA14 KM054243

TaupsbAGenBank4.sqn BAG01 KM054244

TaupsbAGenBank4.sqn BAG02 KM054245

TaupsbAGenBank4.sqn BAG04 KM054246

TaupsbAGenBank4.sqn BAG05 KM054247

TaupsbAGenBank4.sqn BAG07 KM054248

TaupsbAGenBank4.sqn BAG08 KM054249

TaupsbAGenBank4.sqn BAG09 KM054250

TaupsbAGenBank4.sqn BAG10 KM054251

TaupsbAGenBank4.sqn BAG11 KM054252

TaupsbAGenBank4.sqn BAG13 KM054253

TaupsbAGenBank4.sqn BAG15 KM054254

TaupsbAGenBank4.sqn BAG16 KM054255

TaupsbAGenBank4.sqn BAG17 KM054256

TaupsbAGenBank4.sqn BAG18 KM054257

TaupsbAGenBank4.sqn BAG19 KM054258

TaupsbAGenBank4.sqn BAG20 KM054259

TaupsbAGenBank4.sqn BAG21 KM054260

TaupsbAGenBank4.sqn BAG27 KM054261

TaupsbAGenBank4.sqn BAR01 KM054262

TaupsbAGenBank4.sqn BAR02 KM054263

TaupsbAGenBank4.sqn BAR03 KM054264

TaupsbAGenBank4.sqn BAR04 KM054265

TaupsbAGenBank4.sqn BAR05 KM054266

TaupsbAGenBank4.sqn BAR09 KM054267

TaupsbAGenBank4.sqn BAR10 KM054268

TaupsbAGenBank4.sqn BAR11 KM054269

TaupsbAGenBank4.sqn BAR12 KM054270

TaupsbAGenBank4.sqn BAR13 KM054271

TaupsbAGenBank4.sqn BAR15 KM054272

TaupsbAGenBank4.sqn BAR16 KM054273

TaupsbAGenBank4.sqn BOD01 KM054274

TaupsbAGenBank4.sqn BOD02 KM054275

TaupsbAGenBank4.sqn BOD03 KM054276

TaupsbAGenBank4.sqn BOD06 KM054277

TaupsbAGenBank4.sqn BOD07 KM054278

TaupsbAGenBank4.sqn BOD08 KM054279

TaupsbAGenBank4.sqn BOD09 KM054280

TaupsbAGenBank4.sqn BOD10 KM054281

TaupsbAGenBank4.sqn BOD11 KM054282

TaupsbAGenBank4.sqn BOD12 KM054283

TaupsbAGenBank4.sqn BOD14 KM054284

TaupsbAGenBank4.sqn BOD15 KM054285

TaupsbAGenBank4.sqn BOD16 KM054286

TaupsbAGenBank4.sqn BOD17 KM054287

TaupsbAGenBank4.sqn BOD18 KM054288

TaupsbAGenBank4.sqn BOD19 KM054289

TaupsbAGenBank4.sqn CAR01 KM054290

TaupsbAGenBank4.sqn CAR02 KM054291

TaupsbAGenBank4.sqn CAR03 KM054292

TaupsbAGenBank4.sqn CAR04 KM054293

TaupsbAGenBank4.sqn CAR05 KM054294

TaupsbAGenBank4.sqn CAR06 KM054295

TaupsbAGenBank4.sqn CAR07 KM054296

TaupsbAGenBank4.sqn CAR08 KM054297

TaupsbAGenBank4.sqn CAR09 KM054298

TaupsbAGenBank4.sqn CAR10 KM054299

TaupsbAGenBank4.sqn CAR11 KM054300

TaupsbAGenBank4.sqn CAR12 KM054301

TaupsbAGenBank4.sqn CAR13 KM054302

TaupsbAGenBank4.sqn CAR14 KM054303

TaupsbAGenBank4.sqn CAR15 KM054304

TaupsbAGenBank4.sqn CAR16 KM054305

TaupsbAGenBank4.sqn CHG01 KM054306

TaupsbAGenBank4.sqn CHG02 KM054307

TaupsbAGenBank4.sqn CHG03 KM054308

TaupsbAGenBank4.sqn CHG04 KM054309

TaupsbAGenBank4.sqn CHG05 KM054310

TaupsbAGenBank4.sqn CHG06 KM054311

TaupsbAGenBank4.sqn CHG07 KM054312

TaupsbAGenBank4.sqn CHG09 KM054313

TaupsbAGenBank4.sqn CHG10 KM054314

TaupsbAGenBank4.sqn CHG11 KM054315

TaupsbAGenBank4.sqn CHG12 KM054316

TaupsbAGenBank4.sqn CHG13 KM054317

TaupsbAGenBank4.sqn CHG14 KM054318

TaupsbAGenBank4.sqn CHG15 KM054319

TaupsbAGenBank4.sqn CHG16 KM054320

TaupsbAGenBank4.sqn FAT01 KM054321

TaupsbAGenBank4.sqn FAT02 KM054322

TaupsbAGenBank4.sqn FAT03 KM054323

TaupsbAGenBank4.sqn FAT04 KM054324

TaupsbAGenBank4.sqn FAT05 KM054325

TaupsbAGenBank4.sqn FAT06 KM054326

TaupsbAGenBank4.sqn FAT11 KM054327

TaupsbAGenBank4.sqn FAT13 KM054328

TaupsbAGenBank4.sqn FAT14 KM054329

TaupsbAGenBank4.sqn FAT15 KM054330

TaupsbAGenBank4.sqn FAT16 KM054331

TaupsbAGenBank4.sqn FAT20 KM054332

TaupsbAGenBank4.sqn FAT21 KM054333

TaupsbAGenBank4.sqn FAT22 KM054334

TaupsbAGenBank4.sqn FAT23 KM054335

TaupsbAGenBank4.sqn FAT31 KM054336

TaupsbAGenBank4.sqn FAT33 KM054337

TaupsbAGenBank4.sqn FAT35 KM054338

TaupsbAGenBank4.sqn FAT36 KM054339

TaupsbAGenBank4.sqn FAT37 KM054340

TaupsbAGenBank4.sqn FAT38 KM054341

TaupsbAGenBank4.sqn GSV01 KM054342

TaupsbAGenBank4.sqn GSV02 KM054343

TaupsbAGenBank4.sqn GSV03 KM054344

TaupsbAGenBank4.sqn GSV04 KM054345

TaupsbAGenBank4.sqn GSV05 KM054346

TaupsbAGenBank4.sqn GSV06 KM054347

TaupsbAGenBank4.sqn GSV07 KM054348

TaupsbAGenBank4.sqn GSV08 KM054349

TaupsbAGenBank4.sqn NIQ01 KM054350

TaupsbAGenBank4.sqn NIQ02 KM054351

TaupsbAGenBank4.sqn NIQ03 KM054352

TaupsbAGenBank4.sqn NIQ04 KM054353

TaupsbAGenBank4.sqn NIQ05 KM054354

TaupsbAGenBank4.sqn NIQ06 KM054355

TaupsbAGenBank4.sqn NIQ08 KM054356

TaupsbAGenBank4.sqn NIQ10 KM054357

TaupsbAGenBank4.sqn NIQ14 KM054358

TaupsbAGenBank4.sqn NIQ17 KM054359

TaupsbAGenBank4.sqn NIQ19 KM054360

TaupsbAGenBank4.sqn NIQ22 KM054361

TaupsbAGenBank4.sqn NIQ25 KM054362

TaupsbAGenBank4.sqn NIQ26 KM054363

TaupsbAGenBank4.sqn PAN01 KM054364

TaupsbAGenBank4.sqn PAN02 KM054365

TaupsbAGenBank4.sqn PAN03 KM054366

TaupsbAGenBank4.sqn PAN04 KM054367

TaupsbAGenBank4.sqn PAN05 KM054368

TaupsbAGenBank4.sqn PAN06 KM054369

TaupsbAGenBank4.sqn PAN07 KM054370

TaupsbAGenBank4.sqn PAN08 KM054371

TaupsbAGenBank4.sqn PAN09 KM054372

TaupsbAGenBank4.sqn PAN10 KM054373

TaupsbAGenBank4.sqn PAN11 KM054374

TaupsbAGenBank4.sqn PAN12 KM054375

TaupsbAGenBank4.sqn PAN13 KM054376

TaupsbAGenBank4.sqn PAN14 KM054377

TaupsbAGenBank4.sqn PAN15 KM054378

TaupsbAGenBank4.sqn PAN16 KM054379

TaupsbAGenBank4.sqn PNE01 KM054380

TaupsbAGenBank4.sqn PNE02 KM054381

TaupsbAGenBank4.sqn PNE03 KM054382

TaupsbAGenBank4.sqn PNE04 KM054383

TaupsbAGenBank4.sqn PNE05 KM054384

TaupsbAGenBank4.sqn PNE06 KM054385

TaupsbAGenBank4.sqn PNE07 KM054386

TaupsbAGenBank4.sqn PNE08 KM054387

TaupsbAGenBank4.sqn PNE09 KM054388

TaupsbAGenBank4.sqn PNE10 KM054389

TaupsbAGenBank4.sqn PNE11 KM054390

TaupsbAGenBank4.sqn PNE12 KM054391

TaupsbAGenBank4.sqn PNE13 KM054392

TaupsbAGenBank4.sqn PNE14 KM054393

TaupsbAGenBank4.sqn PNE15 KM054394

TaupsbAGenBank4.sqn PNE16 KM054395

TaupsbAGenBank4.sqn POT01 KM054396

TaupsbAGenBank4.sqn POT02 KM054397

TaupsbAGenBank4.sqn POT03 KM054398

TaupsbAGenBank4.sqn POT04 KM054399

TaupsbAGenBank4.sqn POT05 KM054400

TaupsbAGenBank4.sqn POT06 KM054401

TaupsbAGenBank4.sqn POT07 KM054402

TaupsbAGenBank4.sqn POT08 KM054403

TaupsbAGenBank4.sqn POT09 KM054404

TaupsbAGenBank4.sqn POT10 KM054405

TaupsbAGenBank4.sqn POT11 KM054406

TaupsbAGenBank4.sqn POT12 KM054407

TaupsbAGenBank4.sqn POT13 KM054408

TaupsbAGenBank4.sqn POT14 KM054409

TaupsbAGenBank4.sqn POT15 KM054410

TaupsbAGenBank4.sqn POT16 KM054411

TaupsbAGenBank4.sqn PTU01 KM054412

TaupsbAGenBank4.sqn PTU02 KM054413

TaupsbAGenBank4.sqn PTU03 KM054414

TaupsbAGenBank4.sqn PTU04 KM054415

TaupsbAGenBank4.sqn PTU05 KM054416

TaupsbAGenBank4.sqn PTU06 KM054417

TaupsbAGenBank4.sqn PTU07 KM054418

TaupsbAGenBank4.sqn PTU09 KM054419

TaupsbAGenBank4.sqn PTU10 KM054420

TaupsbAGenBank4.sqn PTU11 KM054421

TaupsbAGenBank4.sqn PTU12 KM054422

TaupsbAGenBank4.sqn PTU13 KM054423

TaupsbAGenBank4.sqn PTU14 KM054424

TaupsbAGenBank4.sqn PTU15 KM054425

TaupsbAGenBank4.sqn PTU16 KM054426

TaupsbAGenBank4.sqn PTU18 KM054427

TaupsbAGenBank4.sqn SAF01 KM054428

TaupsbAGenBank4.sqn SAF02 KM054429

TaupsbAGenBank4.sqn SAF03 KM054430

TaupsbAGenBank4.sqn SAF04 KM054431

TaupsbAGenBank4.sqn SAF05 KM054432

TaupsbAGenBank4.sqn SAF06 KM054433

TaupsbAGenBank4.sqn SAF07 KM054434

TaupsbAGenBank4.sqn SAF08 KM054435

TaupsbAGenBank4.sqn SAF09 KM054436

TaupsbAGenBank4.sqn SAF10 KM054437

TaupsbAGenBank4.sqn SAF11 KM054438

TaupsbAGenBank4.sqn SAF12 KM054439

TaupsbAGenBank4.sqn SAF13 KM054440

TaupsbAGenBank4.sqn SAF14 KM054441

TaupsbAGenBank4.sqn SAF15 KM054442

TaupsbAGenBank4.sqn SAF16 KM054443

TaupsbAGenBank4.sqn SCA01 KM054444

TaupsbAGenBank4.sqn SCA02 KM054445

TaupsbAGenBank4.sqn SCA03 KM054446

TaupsbAGenBank4.sqn SCA04 KM054447

TaupsbAGenBank4.sqn SCA05 KM054448

TaupsbAGenBank4.sqn SCA06 KM054449

TaupsbAGenBank4.sqn SCA07 KM054450

TaupsbAGenBank4.sqn SCA09 KM054451

TaupsbAGenBank4.sqn SCA10 KM054452

TaupsbAGenBank4.sqn SCA11 KM054453

TaupsbAGenBank4.sqn SCA12 KM054454

TaupsbAGenBank4.sqn SCA14 KM054455

TaupsbAGenBank4.sqn SCA15 KM054456

TaupsbAGenBank4.sqn SCA16 KM054457

TaupsbAGenBank4.sqn SCA17 KM054458

TaupsbAGenBank4.sqn SDO01 KM054459

TaupsbAGenBank4.sqn SDO02 KM054460

TaupsbAGenBank4.sqn SDO03 KM054461

TaupsbAGenBank4.sqn SEC01 KM054462

TaupsbAGenBank4.sqn SEC02 KM054463

TaupsbAGenBank4.sqn SEC03 KM054464

TaupsbAGenBank4.sqn SEC04 KM054465

TaupsbAGenBank4.sqn STZ01 KM054466

TaupsbAGenBank4.sqn STZ02 KM054467

TaupsbAGenBank4.sqn STZ03 KM054468

TaupsbAGenBank4.sqn STZ04 KM054469

TaupsbAGenBank4.sqn STZ05 KM054470

TaupsbAGenBank4.sqn STZ06 KM054471

TaupsbAGenBank4.sqn STZ07 KM054472

TaupsbAGenBank4.sqn STZ08 KM054473

TaupsbAGenBank4.sqn STZ09 KM054474

TaupsbAGenBank4.sqn STZ10 KM054475

TaupsbAGenBank4.sqn STZ11 KM054476

TaupsbAGenBank4.sqn STZ12 KM054477

TaupsbAGenBank4.sqn STZ13 KM054478

TaupsbAGenBank4.sqn STZ14 KM054479

TaupsbAGenBank4.sqn STZ15 KM054480

TaupsbAGenBank4.sqn STZ16 KM054481

TaupsbAGenBank4.sqn STZ17 KM054482

TaupsbAGenBank4.sqn SUM01 KM054483

TaupsbAGenBank4.sqn SUM02 KM054484

TaupsbAGenBank4.sqn SUM03 KM054485

TaupsbAGenBank4.sqn SUM04 KM054486

TaupsbAGenBank4.sqn SUM05 KM054487

TaupsbAGenBank4.sqn SUM06 KM054488

TaupsbAGenBank4.sqn SUM07 KM054489

TaupsbAGenBank4.sqn SUM08 KM054490

TaupsbAGenBank4.sqn SUM09 KM054491

TaupsbAGenBank4.sqn SUM10 KM054492

TaupsbAGenBank4.sqn SUM11 KM054493

TaupsbAGenBank4.sqn SUM12 KM054494

TaupsbAGenBank4.sqn SUM13 KM054495

TaupsbAGenBank4.sqn SUM14 KM054496

TaupsbAGenBank4.sqn VIB01 KM054497

TaupsbAGenBank4.sqn VIB02 KM054498

TaupsbAGenBank4.sqn VIB03 KM054499

TaupsbAGenBank4.sqn VIB04 KM054500

TaupsbAGenBank4.sqn VIB05 KM054501

TaupsbAGenBank4.sqn VIB06 KM054502

TaupsbAGenBank4.sqn VIB07 KM054503

TaupsbAGenBank4.sqn VIB08 KM054504

TaupsbAGenBank4.sqn VIB09 KM054505

TaupsbAGenBank4.sqn VIB10 KM054506

TaupsbAGenBank4.sqn VIB11 KM054507

TaupsbAGenBank4.sqn VIB12 KM054508

TaupsbAGenBank4.sqn VIB13 KM054509

TaupsbAGenBank4.sqn VIB14 KM054510

TaupsbAGenBank4.sqn VIB15 KM054511

TaupsbAGenBank4.sqn VIB16 KM054512
